# Identification and structural insights into RNA motifs targeted by a CAG repeat DNA-binding small molecule

**DOI:** 10.1039/d5sc05255f

**Published:** 2025-08-21

**Authors:** Qingwen Chen, Aina Fujiwara, Kazuhiko Nakatani, Gota Kawai, Asako Murata

**Affiliations:** a Department of Regulatory Bioorganic Chemistry, SANKEN, The University of Osaka 8-1 Mihogaoka, Ibaraki Osaka 567-0047 Japan murata.asako.012@m.kyushu-u.ac.jp; b Department of Life Science, Faculty of Advanced Engineering, Chiba Institute of Technology Tsudanuma 2-17-1, Narashino Chiba 275-0016 Japan; c Department of Material Sciences, Faculty of Engineering Sciences, Kyushu University 6-1 Kasuga-koen Kasuga Fukuoka 816-8580 Japan

## Abstract

RNA-targeting small molecules have the potential to modulate RNA function and offer possible applications in drug development. However, their molecular recognition mechanisms remain poorly understood due to limited structural insights. To advance our understanding of how these small molecules interact with their target RNAs, it is essential to identify and analyze RNA–small molecule complexes that can serve as models for structural studies. In this study, we identified novel RNA motifs that bind selectively to the small molecule naphthyridine–azaquinolone (NA), previously known to interact with CAG/CAG motif in DNA. Using different methods, including surface plasmon resonance (SPR), thermal melting, and cold-spray ionization mass spectrometry, we investigated the interaction between NA and the RNA motifs. Furthermore, we determined the solution structure of the NA–RNA complex, revealing a distinct binding mode from its DNA interaction. The findings in this study provide molecular-level insight into RNA–small molecule recognition and highlight the potential of NA as a scaffold for developing RNA-targeting small molecules.

## Abstract 2

Novel RNA motifs binding to a small molecule NA were identified *via in vitro* selection. A conserved internal loop was characterized, and its interaction with NA was investigated by NMR, SPR, UV melting, and MS, revealing a unique binding mode.

## Graphical abstract

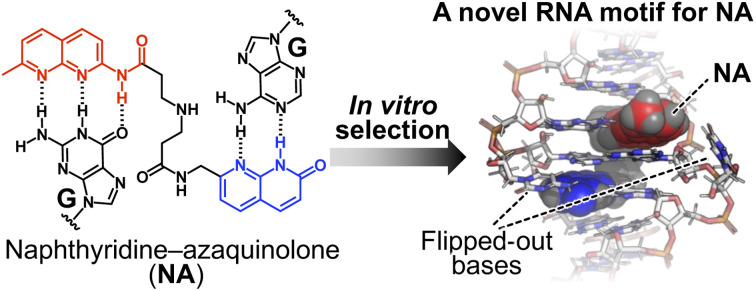

## Introduction

With the identification of its diverse functions, RNA has emerged as a promising target for therapeutic exploration.^1–3^ Since developing new drugs that target proteins has become more challenging, RNA has attracted attention as a molecular target because of its abundance in cells and functional variety.^4–6^ Moreover, targeting RNA will provide opportunities to target undruggable proteins and permit therapeutic intervention in genetic disorders, including neurodegenerative diseases that lack effective treatments.^7–10^ In response to the increasing interest in modulating RNA function, particularly in disease-related contexts, small molecules capable of binding to RNA have attracted considerable attention from researchers in various research fields. Since RNA structure plays a crucial role in RNA function, researchers have explored small molecules that target a specific RNA structure through screening and molecular design. These small molecules target a wide variety of RNAs, including ribosomal RNA,^11,12^ miRNA precursors,^13–15^ riboswitches,^16,17^ and RNA repeats,^18–21^ and other functional RNAs,^22^ all of which have the ability to form distinct tertiary structures.

Despite the appeal of RNA as a drug target and of small molecules as therapeutic modalities, progress in developing RNA-targeted small molecules has been slower than that in protein-targeted approach. This is mainly due to the limited examples of small molecules known to bind to RNA and technical challenges in analyzing the structures of RNA–small molecule complexes. Consequently, the rational design of small molecules targeting RNA remains challenging.

To facilitate the rational design of small molecules targeting specific RNAs, it is helpful to have examples of RNA–small molecule interactions and investigate the mechanisms by which these molecules bind their target RNAs.

**Fig. 1:**
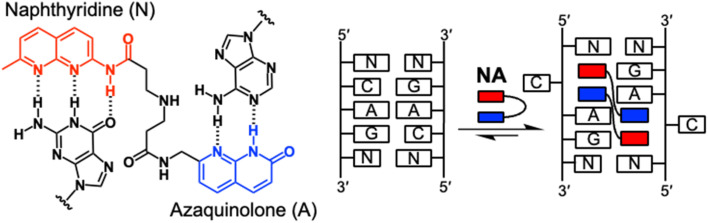
Chemical structure of naphthyridine–azaquinolone (NA) and its hydrogen bonding with nucleobases. NA binds to the adenine–adenine (A–A) mismatch within the 5′-CAG-3′/5′-CAG-3′ (CAG/CAG) motif in a DNA duplex with 2 : 1 binding stoichiometry. The red rectangle indicates the naphthyridine moiety, and the blue rectangle indicates the azaquinolone moiety.

Previously, we developed small molecules that can bind to non-canonical structures in DNA and RNA, especially to mismatched base pairs.^21,24–28^ One of these small molecules, naphthyridine–azaquinolone (NA), selectively binds to adenine–adenine (A–A) mismatches within the 5′-CAG-3′/5′-CAG-3′ (referred to as CAG/CAG) motif in a DNA duplex with 2 : 1 binding stoichiometry (Fig. 1).^29^ Employing this selective binding capability of NA, we effectively induced contractions of the expanded CAG repeats in cellular and mouse models of Huntington's disease.^23^ NA exerts its effect by binding to long slipped-out hairpins formed by expanded CAG repeats during HTT transcription. This binding event interferes with the repair of CAG slip-outs by repair proteins, thereby leading to contraction of the repeat tract. The success observed with NA is attributable to its remarkable selectivity for CAG/CAG motifs within DNA. However, NA exhibits minimal affinity for CAG/CAG motifs in RNA. Our recent findings indicated that the dimerization of NA enhances its binding affinity for CAG/CAG motifs in RNA,^30^ highlighting its diverse binding modes. Therefore, investigating the mechanisms underlying NA's interaction with RNA and its ability to target motifs beyond CAG/CAG motifs is crucial for broadening its application as a modulator of nucleic acid structures and functions, with important implications for drug development with minimal side effects. In the present study, we identified new RNA motifs for NA binding using an *in vitro* selection technique and evaluated the binding affinity of NA for RNA and the mode of NA–RNA complex formation using surface plasmon resonance (SPR), thermal melting, and cold-spray ionization mass spectrometry. Additionally, to investigate the mechanisms by which NA binds their target RNA motifs, we performed a detailed structural analysis of the NA–RNA complex by NMR spectroscopy.

## Results and discussion

### *In vitro* selection identified an RNA motif specific for NA

**Fig. 2:**
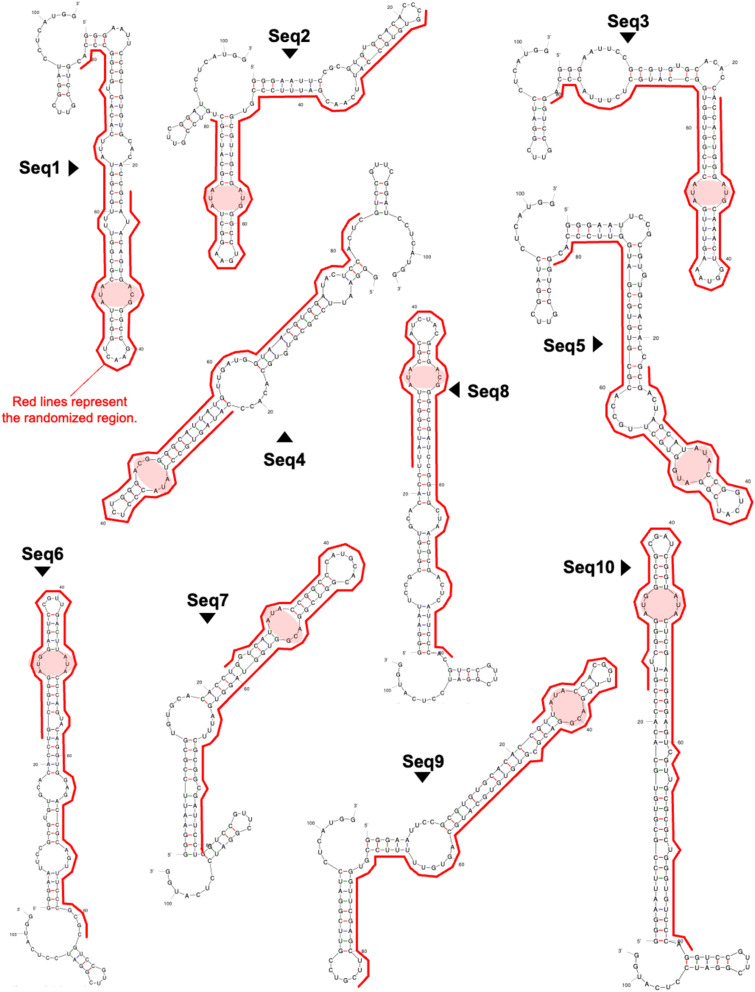
Predicted secondary structures of the 10 selected clones. The structures with the lowest minimal free energy are presented. The red lines represent the randomized region. The 3 base × 3 base internal loops are highlighted with red circles.

We performed *in vitro* selection^31^ to isolate RNA aptamers specifically bound by NA. *In vitro* selection, or SELEX, has proven to be a powerful technique for discovering novel binding species (aptamers) for a given molecular target from a pool of random sequences. We prepared an RNA library containing a random 60-nucleotide region flanked by primer-binding regions at the 5′ and 3′ ends and applied it in the selection experiment. We examined the enrichment of RNA aptamers with high affinity for NA during *in vitro* selection process through UV measurements of the RNA libraries before and after loading onto the NA-immobilized resin, as well as by SPR measurements of the RNA libraries against an NA-immobilized SPR sensor chip. The affinity of NA for the RNA library began to increase after the 6th round and reached saturation by the 10th round (Fig. S1). The 12th and 13th DNA libraries were generated from the RNA molecules that had bound to NA following the 11th and 12th selection rounds, and subsequently, they were cloned for sequencing. Forty-eight clones were isolated from each DNA library and sequenced by the Sanger method. Sequence analysis identified multiple clones that appeared more than twice in either the 12th or 13th DNA library and appeared more than twice across both the 12th and 13th DNA libraries (Table 1, the DNA sequences were converted into RNA sequences). A single nucleotide insertion was observed in a few clones (Seq1 and Seq4). The predominant clone in the 13th DNA library was Seq1, accounting for 4 of 48 isolated clones. Seq2 predominated in the 12th DNA library (3 of 48 isolated clones). However, it was not detected among the 48 clones isolated from the 13th DNA library. The enrichment of these clones was further confirmed by next-generation sequencing, which identified two new clones (Seq9 and Seq10, Table S1) among the top seven enriched sequences. Although the enrichment of multiple sequence clones was observed following *in vitro* selection, no consensus sequence was found among the ten clones (Seq1–Seq10). Therefore, we compared their secondary structures and searched for potential motifs within these structures essential for binding to NA. The secondary structures of Seq1–Seq10 were predicted using the mFold program (Fig. 2).

**Table 1:** RNA clones obtained by *in vitro* selection

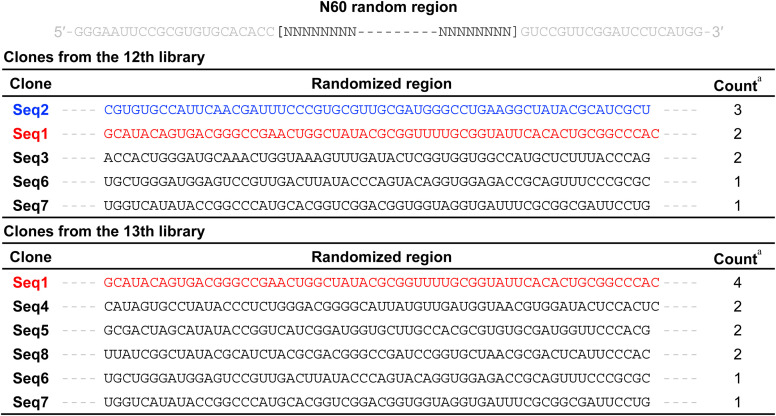

a The count of clones among the 48 isolated clones.

**Fig. 3:**
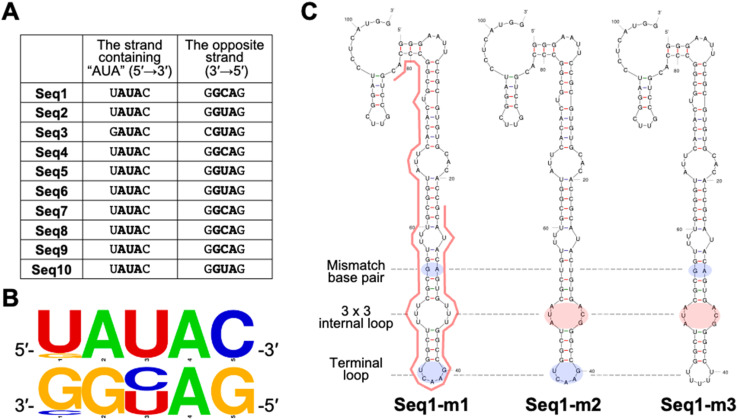
Motif identification. (A) The sequences of the 3 × 3 internal loops. (B) The sequence logo of the 3 × 3 internal loop with flanking base pairs. The logo was generated using the WebLogo online tool (https://weblogo.berkeley.edu/logo.cgi). (C) The designed mutants of Seq1 for binding analysis.

All clones were predicted to form a stem–loop structure within the randomized region (highlighted by the red line in Fig. 2), and each clone contained at least one internal loop. Upon closer examination of these internal loops, a 3 base × 3 base internal loop (3 × 3 internal loop) was consistently found near the terminal loop. Notably, these 3 × 3 internal loops all shared a 5′-AUA-3′/5′-AYG-3′ (Y = C or U) sequence motif flanked by GU or CG base pairs (Fig. 3A and B and Table S2). In addition to the optimal structures of Seq1–Seq10 shown in Fig. 2, this same motif was also present in their suboptimal secondary structures, further underscoring its structural significance in these RNA clones (Fig. S2). After identifying this characteristic motif in Seq1–Seq10, we revisited the secondary structures of each of the 48 clones from the 12th and 13th DNA libraries. Among these clones, 41 of 48 clones in the 12th DNA library and 39 of 48 clones in the 13th DNA library contained a similar 3 × 3 internal loop with a 5′-ANA-3′/5′-ABG-3′ (N = A, U, G, or C; B = U, G, or C) motif (Fig. S3, Tables S2 and S3). These findings strongly indicate the significance of this 3 × 3 internal loop in NA binding. Thus, we examined the binding properties of the clones possessing the 3 × 3 internal loop.

### The internal loop containing a 5′-AUA-3′/5′-AYG-3′ motif is crucial for binding to NA

**Fig. 4:**
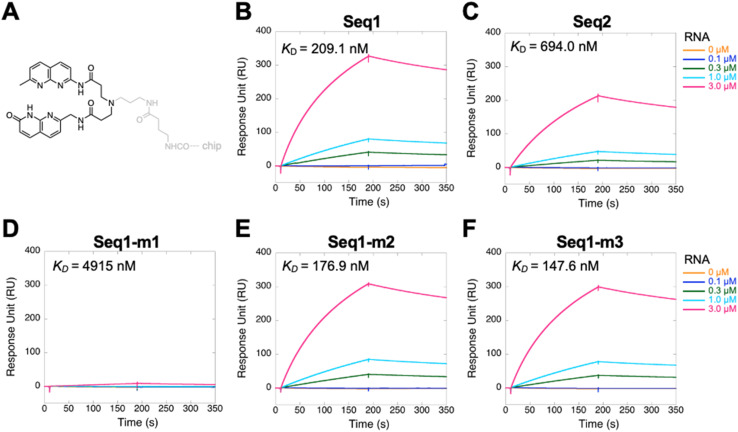
Binding analysis between RNAs and NA by SPR. (A) Schematic illustration depicting the immobilization of NA onto the SPR sensor chip *via* a methylene linker. SPR measurements of the binding of (B) Seq1, (C) Seq2, (D) Seq-m1, (E) Seq-m2, and (F) Seq1-m3 with NA. Different RNA concentrations are represented by different colors. The obtained SPR data were fitted to a 1 : 1 binding model to determine the kinetic parameters and *K*_D_ values.

To validate the interaction between NA and the clones identified by *in vitro* selection, we analyzed their binding using SPR. We specifically selected clones Seq1 and Seq2 for this analysis because they exhibited the highest enrichment during the later rounds of *in vitro* selection. Both Seq1 and Seq2 exhibited significant binding responses on the NA-immobilized SPR sensor surface (Fig. 4A). The dissociation constant (*K*_D_) determined from the kinetic constants was 209.1 nM for Seq1 and 694.0 nM for Seq2 (Fig. 4B and C). In addition, we investigated the binding of these clones with two other small molecules structurally similar to NA to assess their selectivity for NA (Fig. S4). Seq1 and Seq2 showed significantly weaker SPR signals on the naphthyridine carbamate dimer (NCD)-immobilized sensor surface than on the NA-immobilized sensor surface. However, the *K*_D_ values of the complexes determined from the kinetic constants were similar to those obtained for the complexes with NA. The binding capability of NCD could be attributable to the high similarity of the chemical structure between NCD and NA, as both contain a 1,8-naphthyridine moiety in their structures. On the other hand, Seq1 and Seq2 exhibited minimal binding signals on the chip immobilized with the restricted naphthyridine dimer (RND). Both NCD and RND have two naphthyridine moieties, but they differ in the length of the linker connecting the naphthyridine rings and the substituents on naphthyridine. Based on these results, it is evident that Seq1 and Seq2 exhibited the highest affinity for NA. Moreover, they seem capable of distinguishing the structural differences among the three molecules NA, NCD, and RND.

Having confirmed the binding between NA and the enriched clones obtained by *in vitro* selection, we next examined whether their characteristic 3 × 3 internal loop is crucial for NA binding. To investigate this, we designed three Seq1 mutants, each featuring an alteration of one of the three secondary structural motifs within the randomized region of Seq1 that could potentially serve as a binding target for NA (Fig. 3C). In Seq1-m1, all nucleobases comprising the 3 × 3 internal loop (5′-AUA-3′/5′-ACG-3′) were replaced with uracil, which likely exhibits the least affinity for the NA molecule. In Seq1-m2 and Seq1-m3, the nucleobases in the mismatched base pair and terminal loop, respectively, were replaced with uracil. The binding properties of these mutants with NA were examined by SPR. Replacement of the nucleobase forming the 3 × 3 internal loop with uracil significantly reduced the binding affinity of Seq1-m1 for NA (Fig. 4D). In contrast, altering the nucleobases within the other secondary structural motifs, as performed in Seq1-m2 and Seq1-m3, did not affect their ability to bind to NA (Fig. 4E and F). These observations clearly indicate that the 3 × 3 internal loop is the motif responsible for the binding of Seq1 with the NA molecule.

### A simple RNA hairpin with the ACG/AUA motif is essential for binding to NA

**Fig. 5:**
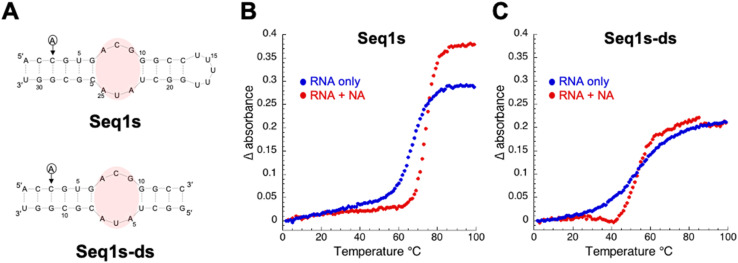
Thermal melting profiles for the interactions of NA with the short versions of Seq1 sequence. (A) Sequences and secondary structures of Seq1s and Ses1s-ds. Thermal melting profiles of (B) Seq1s and (C) Seq1s-ds in the presence and absence of 10 μM NA.

To identify the minimal sequence of Seq1 capable of binding to NA and further analyze the binding mode between the 3 × 3 internal loop in Seq1 and NA, we designed a short version of Seq1 (Seq1s) and its corresponding double-stranded RNA (Seq1s-ds) (Fig. 5A). In both RNA molecules, the adenine at the 3 position was changed to cytosine so that the newly formed CG pair can compensate the destabilization of the RNA structure caused by shortening the sequence. In Seq1s, the terminal loop sequence of Seq1 was changed from 5′-GAACU-3′ to 5′-UUUUU-3′ because this change did not significantly affect the binding ability of Seq1 to NA (Fig. 4F). SPR analysis revealed that Seq1s could bind to NA, although to a lesser extent than the parental Seq1 (Fig. S5). On the other hand, Seq1s-ds exhibited much lower binding responses to the NA-immobilized sensor chip within the same concentration range. This could be attributable to the lower thermal stability of Seq1s-ds than that of Seq1s, as well as the less efficient formation of the conserved structures.

We next investigated whether the binding of NA stabilizes Seq1s and Seq1s-ds by UV-thermal melting experiments (Fig. 5B and C). NA increased the thermal stability of both Seq1s and Seq1s-ds, with a more pronounced effect observed for the former. In the presence of 10 μM NA, an increase in the melting temperature (*T*_m_) from 66.9 ± 2.0 °C to 72.1 ± 1.5 °C (Δ*T*_m_ = 5.2 °C) was observed for Seq1s. In contrast, *T*_m_ increased only slightly (Δ*T*_m_ = 1.8 °C) from 52.9 ± 0.3 °C to 54.7 ± 0.6 °C for Seq1s-ds in the presence of NA. In both cases, the melting curves became steeper in the presence of NA, suggesting the influence of NA binding on the thermal melting profiles of these RNAs. The binding between Seq1s and NA was demonstrated by both SPR and thermal melting analysis. We selected Seq1s as the minimal sequence for further experiments, considering the simplicity of the analysis.

Our previous study demonstrated that NA binds to its target CAG/CAG motif in a DNA duplex with 2 : 1 binding stoichiometry. Given that the RNA motifs specific for NA obtained by *in vitro* selection were distinct from the target motif of NA in DNA, we sought to determine the binding stoichiometry of NA to the target RNA motifs. Therefore, we investigated the stoichiometry of NA binding with the 3 × 3 internal loop featuring 5′-AUA-3′/5′-AYG-3′ by cold-spray ionization time-of-flight mass spectrometry (CSI-TOF MS). The addition of excess NA (40 μM) to Seq1s (5 μM) led to the production of distinct ions at *m*/*z* of 1725.41 and 2070.57 corresponding to 6− and 5− ions, respectively (Fig. S6). This result suggests that NA most likely binds to Seq1s with 1 : 1 stoichiometry.

### Search for the identified motif in non-coding RNAs

To explore potential cellular RNA targets of NA, we systematically analyzed the secondary structure of human non-coding RNAs (ncRNAs). A dataset containing 203 745 ncRNA sequences was obtained from Ensembl release 114,^32^ and local secondary structures were predicted using RNAfold (ViennaRNA package 2.7).^33^ For sequences ≤500 nucleotides (nts), full-length folding predictions were performed. For longer transcripts, a sliding window approach (500 nt window with 100 nt overlap) was applied to capture local structural features while accommodating the complexity of long ncRNAs (Fig. S7A). The resulting secondary structures, represented in dot-bracket notation, were computationally screened for the 3 × 3 internal loops with the sequences identified in the SELEX experiment: 5′-GACGG-3′/5′-UAUAC-3′ and 5′-GAUGG-3′/5′-UAUAC-3′. An in-house Python script was developed to search for these motifs within the predicted RNA structures. Using the script, we found that the internal loop motifs are present in the predicted secondary structure of nine ncRNA molecules (Fig. S7B). While the functions of most of these ncRNAs remain uncharacterized, one notable example is PROX1 antisense RNA 1 (PROX1-AS1), a long non-coding RNA (lncRNA) implicated in the regulation of the PROX1 gene (Fig. S7C). PROX1 is a key transcription factor that governs muscle cell differentiation, fiber-type identity, and actively represses cell fate plasticity to maintain hepatocyte identity and prevent liver tumorigenesis, whereas its regulator, the lncRNA PROX1-AS1, promotes tumor progression by modulating gene expression *via* the miR-326/FBXL20 axis.^34–36^ This finding suggests that NA-binding motifs may exist in biologically relevant RNA contexts. Although experimental validation of NA binding to these endogenous RNAs and assessment of downstream biological effects in cellular systems were beyond the scope of this exploratory study, the results generate experimentally testable hypotheses for subsequent functional studies, as well as provide a foundation for future investigations into the transcriptome-wide relevance of NA-interacting RNA motifs and their potential therapeutic implications.

### NMR analysis revealed the unique binding mode between NA and its target RNA

To better understand the mechanism of binding between NA and the 3 × 3 internal loop in the RNA clones obtained by *in vitro* selection, we analyzed the interaction between NA and a model RNA sequence by NMR spectroscopy. In these analyses, we used the model RNA Seq1s-4U, in which the terminal loop of Seq1s was changed from 5′-UUUUU-3′ to 5′-UUUU-3′.

**Fig. 6:**
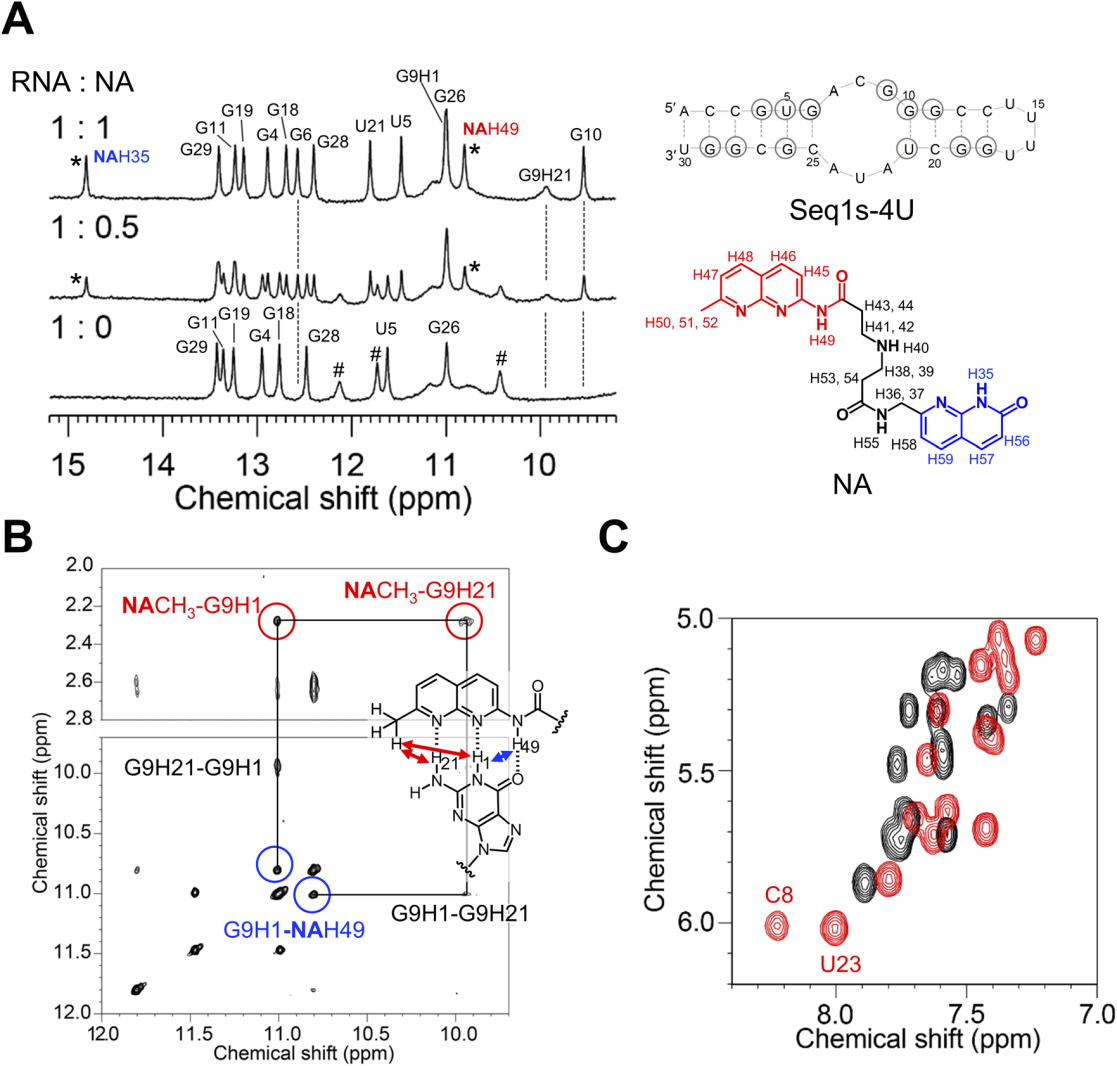
NMR structural analysis of the NA–Seq1s-4U complex. (A) Changes in the imino proton spectra of Seq1s-4U upon the addition of NA. Asterisks (*) denote the signals of NA complexed with Seq1s-4U. #: these signals are due to G6, G10 or U21. (B) NOESY spectra of the complex between NA and Seq1s-4U. Correlations between amino/imino hydrogens and the methyl group (red circles), as well as between amide and imino hydrogens (blue circles), are presented with the assignment of hydrogens. (C) Partial two-dimensional HOHAHA spectra of Seq1-4U in the absence (black) and presence (red) of NA.

NA solution was added to Seq1s-4U dissolved in phosphate buffer containing 5% D_2_O, and the NMR spectra were recorded. Partial signals from the imino protons of Seq1s-4U were assigned using the NOESY spectra. As the molar ratio of NA to RNA increased, the signal intensities of the imino protons from the unbound RNA decreased, whereas new signals from the NA–RNA complex appeared (Fig. 6A). The signals from the imino protons of G6, G9, and G10, along with the amino proton of G9, became detectable only upon the addition of NA, suggesting an interaction between NA and the 3 × 3 internal loop region. In addition to the signals from the RNA molecules, signals from the amide protons of NA (NAH35 and NAH49, Fig. 6A), which potentially form hydrogen bonds with the RNA, were also observed. At a molar ratio of 1 : 1, only the signals from the NA–RNA complex were observed, indicating that NA binds to Seq1s-4U with 1 : 1 binding stoichiometry, which is consistent with the result of CSI-TOF MS analysis. Note that signals from both unbound and bound RNAs were detected at an RNA–NA molar ratio of 1 : 0.5, indicating that the NA–RNA complex was stable under the experimental conditions. Inter-molecular NOEs between the naphthyridine moiety of NA and G9 were observed (Fig. 6B). Correlations between the amino hydrogen and methyl group (red circle) and amide and imino hydrogens (blue circle) were detected. Notably, the signal at 14.81 ppm shows strong NOE with a signal at 8.06 ppm which was assigned to A24H2, indicating the formation of azaquinolone-A24 base pair as shown in Fig. S7A. Signal assignment for the NA–RNA complex was confirmed by residue-specific stable isotope labeling for U23 and A24 (Fig. S7B). NOEs were observed between A7 and G9, as well as between A22 and A24 (Fig. S6C, orange). Additionally, an NOE between A7 and A24 was detected (Fig. S7C, purple). These findings indicate the spatial proximity of these nucleotide pairs within the NA–RNA complex. NMR signals from nucleobases located within and near the 3 × 3 internal loop were shifted upon NA addition, as indicated by chemical shift differences between free RNA and the RNA–NA complex, with those from C8 and U23 exhibiting significant downfield shifts (Fig. S8 and Table S4). Comparison of the HOHAHA spectrum of RNA in the presence (red) and absence (black) of NA revealed a shift in most proton signals from the RNA upon NA addition, indicating a conformational change (Fig. 6C). Consistent with the earlier observation, a significant shift was detected for C8 and U23. These results suggest that these nucleobases are flipped out of the RNA stack upon NA binding. The flipping of unpaired bases from the DNA/RNA helix was observed during the binding of our mismatch-binding ligands.^29,37^

**Fig. 7:**
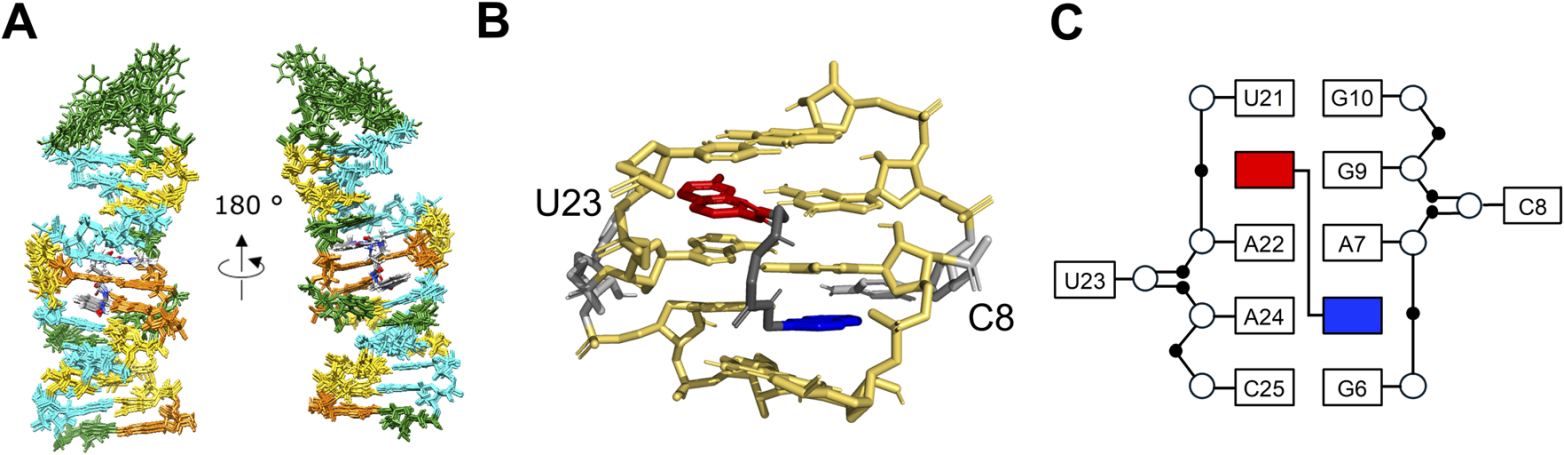
Solution structures of the NA–Seq1s-4U complex. (A) Superposition of the ten lowest total energy structures of the complex between NA and Seq1s-4U. (B) Close-up structure of the 5′-AUA-3′/5′-ACG-3′ motif within Seq1s-4U bound with NA and (C) its structure pattern diagram.

Finally, we determined the solution structure of the NA–RNA complex. The superposition of the ten lowest energy structures is presented in Fig. 7A, and the structural statistics are summarized in Table S5. The structures of the NA–Seq1s-4U complex were well defined, and close-up views of the minimized average structure of the binding site of NA are presented in Fig. 7B. As illustrated in Fig. 7C, the naphthyridine and azaquinolone moieties formed base pairs with G6 and A24, respectively, and A7 and A22 were stacked between the naphthyridine–G6 and azaquinolone–A24 base pairs. As a result, A7 and A22 form a base pair between the Watson–Crick edge of A7 and the Hoogsteen edge of A22 (Fig. S9).

In this study, we isolated RNA aptamers that bind specifically to the small molecule NA. Among these aptamers, we identified a characteristic 3 × 3 internal loop with the 5′-AUA-3′/5′-AYG-3′ (Y = C or U) motif. Seq1, the most enriched aptamer, strongly bound to NA with a submicromolar dissociation constant, as demonstrated by SPR measurement. The shorter version of Seq1 Seq1s, maintained binding affinity for NA, although its *K*_D_ was three to four times higher than that observed for the Seq1–NA interaction. The 3 × 3 internal loop with 5′-AUA-3′/5′-AYG-3′ was found to be crucial for NA's binding, however, the binding affinity can be influenced by surrounding sequences and structural context. As part of the discussion, we additionally designed eight Seq1s mutants (m1–m8) to compare the flanking base pair sequence and its role in NA recognition. The flanking base pairs of Seq1s were replaced with the different base pairs not found in Seq1–Seq10 (Fig. S10A) and examined their binding to the NA-immobilized SPR sensor chip (Figs. S10B and C). When comparing the response units at steady state (4 s before the end of analyte injection), the response signals of all eight RNA mutants were weaker than that of the parental RNA. The mutant m1, in which the G10–U22 base pair was replaced with U10–G22, produced a much lower response on the NA-immobilized sensor surface than the other mutants. The SPR signals varied according to the flanking base pairs, although no significant relationship was observed between the intensity of the binding signal and the base-pair types (Fig. S10C). It is noted that the flanking base pairs may influence NA binding through alterations in stacking and/or electrostatic interactions, and that further structural studies, including MD simulations, may be necessary to understand the binding mechanism.

Finally, we determined the solution structure of the NA–RNA complex using NMR spectroscopy, which revealed the interactions important for its specificity. Formation of hydrogen bonds between NA and the adenine and the guanine within the 3 × 3 internal loop (5′-AUA̲-3′/5′-ACG̲-3′, underlined bases) is accompanied by the flipping of U out of the RNA stack. Additionally, the other two adenine bases in the 3 × 3 internal loop (5′-A̲UA-3′/5′-A̲CG-3′, underlined bases) formed a non-canonical base pair stabilized by hydrogen bonding between the Watson–Crick and Hoogsteen edges. This particular structural arrangement may explain why these adenines were preferentially selected through the *in vitro* selection process. As illustrated in Fig. S11, replacing the adenine opposite to the guanine in the 3 × 3 internal loop (5′-A̲UA-3′/5′-AYG-3′, underlined) with the other nucleobases is expected to disrupt the formation of the internal loop. On the other hand, replacing the adenine in the opposite strand (5′-AUA-3′/5′-A̲YG-3′, underlined) with cytosine or guanine does not appear to affect the formation of the 3 × 3 internal loop (Fig. S11). The requirement for adenines in these positions is likely attributable to the aforementioned interaction between two adenines *via* the Watson–Crick and Hoogsteen edges, which could help stabilize the NA–RNA complex.

## Conclusions

Overall, our study identified previously unreported RNA motifs as binding targets for NA. The limited number of known RNA-binding small molecules has been a major challenge in RNA-targeted drug discovery and development. Our demonstration that NA, originally designed as DNA-binding small molecule, also interacts with specific RNA motifs, will expand the chemical space of potential RNA-targeting scaffolds. While the NMR structure provides a detailed characterization of the interaction between the RNA motif and NA, the dynamics of RNA in a cellular environment remains unexplored. Future studies should delve into the interactions of NA with its target RNA motifs and assess its behavior under physiological conditions to broaden the applicability of the results. Although NA is not widely recognized, its well-characterized DNA-binding properties make it a valuable model for studying RNA–small molecule interactions. Identifying RNA motifs that selectively bind to NA, as well as detailed structural analysis performed in this study, provides insights into molecular recognition principles. NA-binding motifs were found in ncRNAs, with PROX1 antisense RNA 1 (PROX1-AS1) being a notable example, suggesting potential biological relevance. The findings contribute to structure-guided approaches for designing RNA-targeting small molecules and offer valuable data that extend understanding beyond current machine learning capabilities.

## Author contributions

A. M., K. N. and G. K. conceived and designed the experiments. Q. C. performed all *in vitro* experiments, including *in vitro* selection, SPR measurements, *T*_m_ analyses and mass spectrographic analyses. G.K. and A.F. performed structural analysis of RNA–NA complex by NMR spectroscopy. A. M., Q. C., K. N and G. K. wrote the manuscript, and all authors reviewed it.

## Conflicts of interest

The authors declare no conflict of interest.

## Supplementary Material

SC-OLF-D5SC05255F-s001

## Data Availability

Solution structures of the complex of naphthyridine–azaquinolone (NA) and an Seq1s-4U have been deposited with Protein Data Bank (https://www.rcsb.org) under accession number 8ZNQ. Upon a reasonable request, the authors will provide the raw data, additional information and materials. The requests should be addressed to A. M. The data supporting this article have been included as part of the SI. Details of materials and methods, synthesis and characterization of the compounds, as well as supporting data for sequencing, SPR, ESI, NMR analyses, are provided in the SI. See DOI: https://doi.org/10.1039/d5sc05255f.
