# Supplementary material for: Identification and structural insights into RNA motifs targeted by a CAG repeat DNA-binding small molecule
Source: Chem Sci. 2025 Aug 21;16(36):16819–28. doi: 10.1039/d5sc05255f (PMC12368606; doi:10.1039/d5sc05255f)

Supplementary Information (SI) for Chemical Science.
This journal is © The Royal Society of Chemistry 2025

Supplementary Information for:

Identification and Structural Insights into RNA Motifs Targeted by a CAG Repeat DNA-Binding Small Molecule

Qingwen Chen,[a] Aina Fujiwara,[b] Kazuhiko Nakatani,[a] Gota Kawai,[b] Asako Murata[c,*]

[a]Department of Regulatory Bioorganic Chemistry, SANKEN, The University of Osaka

[b]Department of Life Science, Faculty of Advanced Engineering, Chiba Institute of Technology

[c]Department of Material Sciences, Faculty of Engineering Sciences, Kyushu University

*Corresponding author

Tel; +81-92-583-8845, E-mail; murata.asako.012@m.kyushu-u.ac,jp

Contents

**S1.**	General information	…S2
**S2.**	Materials and Methods	…S2
**Scheme S1.**	Synthetic scheme for the preparation of NA-linker (5)	…S6
**Figure S1.**	Progress and result of the in vitro selection against NA-immobilized resin	…S7
**Figure S2.**	Suboptimal foldings of **Seq1**–**Seq10** predicted by mFold program, and their corresponding ΔG values	…S8
**Figure S3.**	Number of clones containing a the 5'-AUA-3' sequence or the 5'-ABG-3' (B = U, G, or C) sequence within the 3 × 3 internal loop	…S9
**Figure S4.**	SPR analysis of binding of **Seq1** and **Seq2** to different small molecules	…S10
**Figure S5.**	SPR analysis of binding of **Seq1**, **Seq1s**, and **Seq1s-ds** to NA	…S11
**Figure S6.**	CSI-TOF mass spectra of **Seq1s**-NA complex	…S12
**Figure S7.**	Motif search in ncRNA	…S13
**Figure S8.**	Supplementary NMR spectra for characterization of RNA and RNA-NA complex	…S14
**Figure S9.**	Chemical shift differences between free RNA and NA-RNA complex	…S15
**Figure S10.**	Close-up view of the base pairing between A7 and A22 residues	…S16
**Figure S11.**	Effect of the difference in the flanking base pairs of the 3 × 3 internal loop on the interaction with NA	…S17
**Figure S12.**	Predicted secondary structures of mutants of altered 3 × 3 internal loops	…S18
**Table S1.**	The clones identified from the 13th library by next-generation sequencing	…S19
**Table S2.**	The sequences of clones identified from 12th DNA library highlighting the conserved motif	…S20
**Table S3.**	The sequences of clones identified from 13th DNA library highlighting the conserved motif	…S21
**Table S4.**	Chemical shifts of selected protons in free and NA-bound (1:1) RNA	…S22
**Table S5.**	NMR restraints and statistics	…S23
**^{1}H-NMR spectra and Mass spectra of Compound 5 (NA-linker)**		…S24

S1. General information

Reagents and solvents were purchased from standard suppliers and used without further purification. Chemical reactions were monitored by TLC on plates precoated with Merck silica gel 60 F254. Spots were visualized with UV light or ninhydrin. Wakogel C-200 was used for flash chromatography on silica gel. ^{1}H-NMR spectra of compounds synthesized in this study were measured on JEOL JNM-LA400 or LA600 spectrometers. The multiplicity was expressed as follows: s for singlet, d for doublet, t for triplet, quin for quintet, m for multiplet and br for broad. The chemical shifts are expressed in ppm relative to residual solvent as an internal standard and coupling constants (*J* values) are given in Hz.

S2. Materials and Methods

***In vitro* selection of RNA motifs for NA.**

(a) Preparation of NA-immobilized resin: Naphthyridine-azaquinolone (NA) was immobilized to an agarose resin via an amino alkyl linker. The synthetic scheme of NA-linker was shown in **Scheme S1**. NA-linker was immobilized on NHS-activated Sepharose 4 Fast Flow resin (Cytiva) by amino coupling method according to the manufacturer's instructions. Briefly, to NHS-activated Sepharose 4 Fast Flow resin (0.5 mL) was added a solution of NA-linker (50 nmol) in 0.2 M $NaHCO_3$-0.5 M NaCl (pH 8–9) (0.5 mL). After shaking the suspension at room temperature for several hours, the solution was drained, and the resin was further treated with 0.1 M Tris-HCl buffer (1 mL, pH 8.8) for several hours to block unreacted carboxylic groups on the resin. The resulting resin was sequentially washed with 0.1 M Tris-HCl buffer (pH 8.8) and 0.1 M NaOAc-0.5 M NaCl solution (pH 5.2), which was repeated three times. The resin was washed with 20% aqueous EtOH, and three bed volumes of 20% aqueous EtOH was added to the resin to give a 25% slurry resin suspension, which was stored at 4 °C.

(b) Preparation of DNA and RNA pools: The initial double stranded DNA (dsDNA) pool was prepared by PCR amplification of the oligonucleotide containing 60 random nucleotides (5'-GAA TTC CGC GTG TGC ACA CC - N60 - GTC CGT TGG GAT CCT CAT GG-3') as a template with Platinum SuperFi DNA polymerase (ThermoFischer Scientific). The primers used in the PCR amplification were: 5'-GC<u>T AAT ACG ACT CAC TAT A</u>GG GAA TTC CGC GTG TGC ACA CC -3' (forward primer, T7 promoter sequence is underlined) and 5'- CCA TGA GGA TCC GAA CGG AC-3' (reverse primer). The initial dsDNA pool was transcribed in vitro with T7 RNA polymerase (MEGAshortscript T7 Transcription Kit, Ambion) to yield an RNA pool. The reaction mixture was treated with DNase at 37 °C for 30 min to digest the template DNA. The mixture was diluted with RNase free water and passed through a NAP-5 column (Cytiva) to remove unincorporated NTPs. The RNA was precipitated by adding 7.5 M ammonium acetate and isopropanol and pelleted by centrifugation. The pellet was dissolved in annealing buffer (10 mM Tris-HCl, 50 mM NaCl, 100 mM KCl, pH 7.5). The RNA solution was denatured at 80 °C for 3 min and annealed by slow cooling to room temperature. To the annealed RNA was added $MgCl_2$ to the final concentration of 5 mM and used for the next binding step.

(c) Selection step and regeneration of RNA pool: The RNA pool was incubated with 25 μL of the NA-immobilized resin at room temperature for 30 min with vortexing every 10 min. The resin was drained

and washed three times with 100 μL of washing buffer (10 mM Tris-HCl, 100 mM KCl, 50 mM NaCl, 5 mM $MgCl_2$, pH 7.5) to remove the non-binding species. Bound RNAs were eluted three times with NA solution (50 μL, 200 μM) and pooled and precipitated with 0.3 M sodium acetate and ethanol and pelleted by centrifugation. The RNA pellet was dissolved in RNase free water and reverse transcribed with PrimeScript II Reverse Transcriptase (TAKARA) using the reverse primer above. The resulting cDNA was PCR-amplified with the forward and the reverse primers as described above. The DNA templates were transcribed in vitro, and the resulting RNAs were subjected to the next round of selection. After rounds of selection, the enriched libraries (12th- and 13th-dsDNA libraries) were sequenced by a next-generation sequencing (NGS). The same libraries were also cloned and sequenced by Sanger sequencing.

CSI-TOF MS. Samples were prepared by mixing the solution of **Seq1s** RNA (5'-ACC GUG ACG GGC CUU UUU GGC UAU ACG CGG U-3') and NA in 50% methanol in water containing 100 μM ammonium acetate, with final concentrations of 5 μM and 40 μM, respectively. The samples were analyzed by cold spray ionization time-of-flight mass spectrometry (CSI-TOF MS) on a Bruker maXis impact mass spectrometer in the negative mode (orifice 1 voltage = –60 V). Spray temperature was fixed at −10 °C with a sample flow rate of 2 μL/min. The obtained spectrum was analyzed by an open-source mass spectrometry tool mMass (ver. 5.5.0).

***T_m* measurements.** The thermal denaturation profile was recorded on a UV 2700 spectrometer equipped with a TMSPC-8 temperature controller (Shimadzu). The sample solutions were prepared by mixing an RNA sample and NA at the final concentration of 5 μM and 40 μM, respectively, in 10 mM sodium cacodylate buffer (pH 7.0) containing 100 mM NaCl. The absorbance of the sample solutions at 260 nm was monitored from 2 °C to 80 °C with a sample heating rate of 1 °C/min. Melting temperatures (T_m) were calculated by the differential method.

Surface plasmon resonance (SPR) analysis. (a) Immobilization of amino-linker NA to CM5 sensor chip: All immobilizations were performed using 1×HBS-N buffer (10 mM HEPES, 150 mM NaCl, pH 7.4) as running buffer on Biacore T200 instrument (Cytiva) at 25 °C. The carboxy groups of the dextran surface of a sensor chip CM5 (Series S Sensor chip CM5, Cytiva) were activated with NHS/EDC (7 min, 10 μL/min), and 100 mM Amino-dPEG$_4$ acid (QUANTA BIODESIGN) in 1×HBS-N buffer was flowed onto the activated surface at 10 μL/min for 10 min, followed by capping of the residual activated ester with 1 M ethanolamine (5 min, 10 μL/min). The carboxy groups of Amino-dPEG4 acid were activated with NHS/EDC (7 min at 10 μL/min) and 2 mM NA in borate buffer (10 mM sodium tetraborate, pH 9.3) was flowed onto the chip surface (7 min at 10 μL/min). After the coupling, the residual activated ester was capped with 1 M ethanolamine (2 min at 10 μL/min), and the chip surface was washed with 50 mM NaOH to remove the residual ethanolamine. The immobilized amount of NA on the chip surface was 101.8 response units (RU). The SPR sensor chips immobilized with other ligands (NCD and RND) were prepared essentially the same way as described above. The immobilized amount of NCD and RND on the chip surface was 65.3 RU and 622.2 RU, respectively.

Nuclear Magnetic Resonance (NMR) structural analysis: Non-labeled RNA sample was purchased from Hokkaido System Science. The RNA samples were dissolved in 20 mM sodium phosphate buffer (pH 6.5) with 50 mM sodium chloride and 5% D_2O. The RNA sample of 0.26 mM was titrated with 24 mM NA solution. To assign signals of U23 and A24, an RNA sample with 10% $^{13}C/^{15}N$-lebeled U23 and A24 was purchased from Taiyo-Nippon Sanso Corporation to obtain a 0.08 mM solution for NMR measurements. NMR spectra were measured by an AvanceNeo600 spectrometer (Bruker Biospin) at 288 K. The water signal was suppressed by the jump-and-return pulse[1] for 1D imino proton spectra and the 3-9-19 pulse[2] for other experiments. NMR spectra were processed with TopSpin (Bruker Biospin) and analyzed with Sparky.[3] Structures of the RNA-NA complex were calculated by CNS_SOLVE 1.3 (Yale University). For stem region (A1-G6, G10-C13, G18-U21 and C25-U30), dihedral restrains for RNA-A backbone conformation were applied. Based on the NOE intensity between base and ribose H1', the glycosidic bonds were fixed to the *anti*-conformation for all residues. For most residues, the sugar pucker was fixed to the C3'-*endo* form except for U14-U17, A22, U23 for which the sugar pucker was fixed to the C2'-*endo* form based on the H1'- H2' signals in HOHAHA spectra. For C8 and U30, the sugar pucker was not fixed because their H1'- H2' signals in HOHAHA spectra were observed but not strong. Distance restraints for hydrogen bonds were used for the base pairs in the stem region and base pairs between G9 and naphthyridine as well as A24 and azaquinolone. Restraints to maintain the base pair planarity were also used for these base pairs. NOE distance constraints were derived from the cross-peak volumes in NOESY spectra measured with a mixing time of 200 ms. NMR restraints and structure statistics were shown in Supplemental Table S2. Molecular images were prepared with UCSF Chimera.[4]

Synthesis of NA-linker

Compound 3

To a solution of N-(tert-Butoxycarbonyl)-4-aminobutyric acid **2** (335.35 mg, 1.65 mmol) in THF (3 ml) at room temperature was added 1-Amino-3,3-diethoxy propane **1** (220.83 mg, 1.50 mmol). To the mixture was added 1-(3-Dimethylamino propyl)-3-ethylcarbodiimide Hydrochloride (632.74 mg, 3.3 mmol) and 1-hydroxy benzotriazde (catalytic amount, 0.1 eq). The resulting solution was stirred overnight at room temperature. To the reaction mixture was added AcOEt and the aqueous phase as extracted with AcOEt. Combined organic phases were dried over $MgSO_4$, filtered, and concentrated *in vacuo* to give the crude products (84%).

^{1}H-NMR (400 MHz, $CDCl_3$) δ 6.45 (s, 1H), 4.80 (s, 1H), 4.57 (t, J = 5.3 Hz, 1H), 4.12 (q, J = 7.2 Hz, 2H), 3.72-3.64 (m, 3H), 3.55-3.47 (m, 3H), 3.36 (q, J = 6.0 Hz, 3H), 3.16 (q, J = 6.4 Hz, 3H), 2.18 (t, J = 7.1 Hz, 3H), 2.05 (s, 2H), 1.85-1.76 (m, 8H), 1.47-1.40 (m, 9H), 1.28-1.17 (m, 4H)

Compound 4

To a solution of **NA** (200 mg, 0.435 mmol) and **1** (146.07 mg, 0.57 mmol) in MeOH (10 mL) was added acetic acid to adjust pH of the solution to around 6.0, and then added sodium cyanoborohydride (71.07 mg, 1.13 mmol). The solution was stirred for 15 hours at room temperature. To the reaction mixture was added $CHCl_3$, and the organic phase was washed with saturated $NaHCO_3$ and then brine. The organic layer was dried over $MgSO_4$, filtered, and concentrated *in vacuo* to give the

crude products. The residue was purified by column chromatography on silica gel (chloroform/methanol, 50:1 then 30:1, finally 10:1) to give compound **4** in 74% yield.

^{1}H-NMR (600 MHz, $CDCl_3$) δ 8.33-8.41 (1H), 7.97-8.06 (1H), 7.88-7.96 (1H), 7.60-7.69 (1H), 7.53-7.60 (1H), 7.14-7.23 (1H), 7.00-7.07 (1H), 6.53-6.66 (1H), 4.45-4.57 (2H), 3.69-3.78 (4H), 3.47-3.53 (3H), 3.23-3.39 (2H), 3.10-3.21 (4H), 2.91-2.98 (2H), 2.30-2.39 (2H), 1.20-1.29 (6H)

NA-linker (Compound 5)

Compound **4** (30 mg, 0.043 mmol) was dissolved in chloroform (1.5 ml), and 4N HCl(aq)/AcOEt was added dropwise at 0 ˚C. The solution was stirred for 1 hour at 0 ˚C, concentrated in vacuo. The crude product was purified by HPLC to give NA-linker (compound **5**) in 42% yield.

^{1}H-NMR (400 MHz, $CDCl_3$) δ 11.45 (s, 1H), 8.36 (d, J = 8.7 Hz, 1H), 8.17-8.11 (m, 2H), 7.89 (d, J = 8.7 Hz, 2H), 7.61 (d, J = 7.8 Hz, 1H), 7.50 (d, J = 9.6 Hz, 1H), 7.18 (d, J = 8.2 Hz, 1H), 6.98 (d, J = 7.8 Hz, 1H), 6.48 (d, J = 9.6 Hz, 1H), 4.40 (d, J = 5.5 Hz, 2H), 3.26 (q, J = 5.3 Hz, 2H), 3.14 (t, J = 5.7 Hz, 2H), 2.86 (dd, J = 12.4, 4.1 Hz, 2H), 2.80 (t, J = 6.2 Hz, 1H), 2.66 (t, J = 6.6 Hz, 2H), 2.60 (t, J = 8.9 Hz, 6H), 2.06 (d, J = 15.1 Hz, 8H), 1.71 (t, J = 5.5 Hz, 2H). HR-ESIMS calculated for $[M+H]^{+}$: m/e = 602.3198, found: 602.3199, $[M+2H]^{2+}$: m/e = 301.6635, found: 301.6637.

References

1 P. Plateau and M. Gueron, *J Am Chem Soc*, 1982, 104, 7310–7311.

2 M. Piotto, V. Saudek and V. Sklenář, *J Biomol NMR*, 1992, 2, 661–665.

3 T. D. and K. D. G. Goddard, 2008, preprint.

4 E. F. Pettersen, T. D. Goddard, C. C. Huang, G. S. Couch, D. M. Greenblatt, E. C. Meng and T. E. Ferrin, *J Comput Chem*, 2004, 25, 1605–1612.

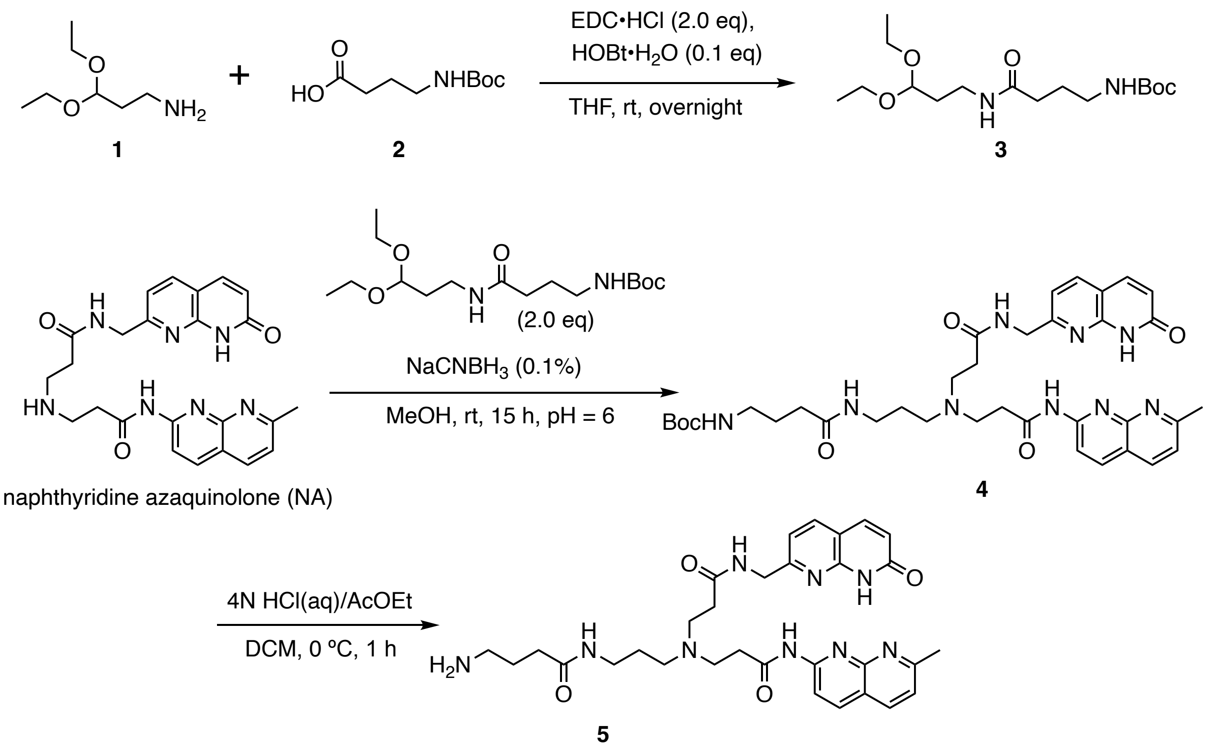

Scheme S1. **Synthetic scheme for the preparation of NA-linker (5).**

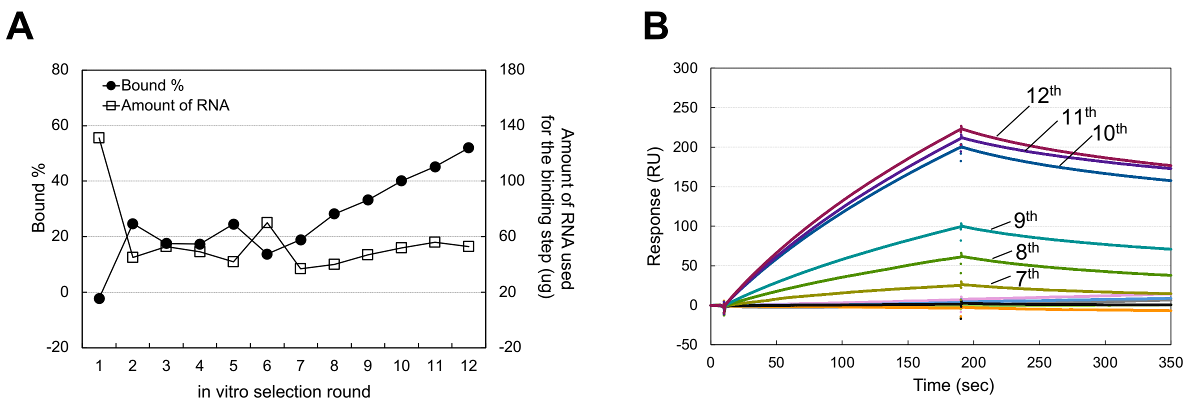

Figure S1. Progress and result of the in vitro selection against NA-immobilized resin. (A) Fraction of RNA that bound to NA at each round of selection. The values of the fraction bound (Bound %) were calculated by the following equation: (Abs260 of the input – Abs260 of the flowthrough) x 100 / Abs260 of the input. (B) SPR analysis of the affinity of RNA pool at each round of selection. SPR sensorgrams of the 1st (gray), 2nd (pink), 3rd (light blue), 4th (light green), 5th (orange), 6th (black), 7th (dark khaki), 8th (dark green), 9th (dark cyan), 10th (dark blue), 11th (indigo), and 12th (dark red) RNA pools are shown.

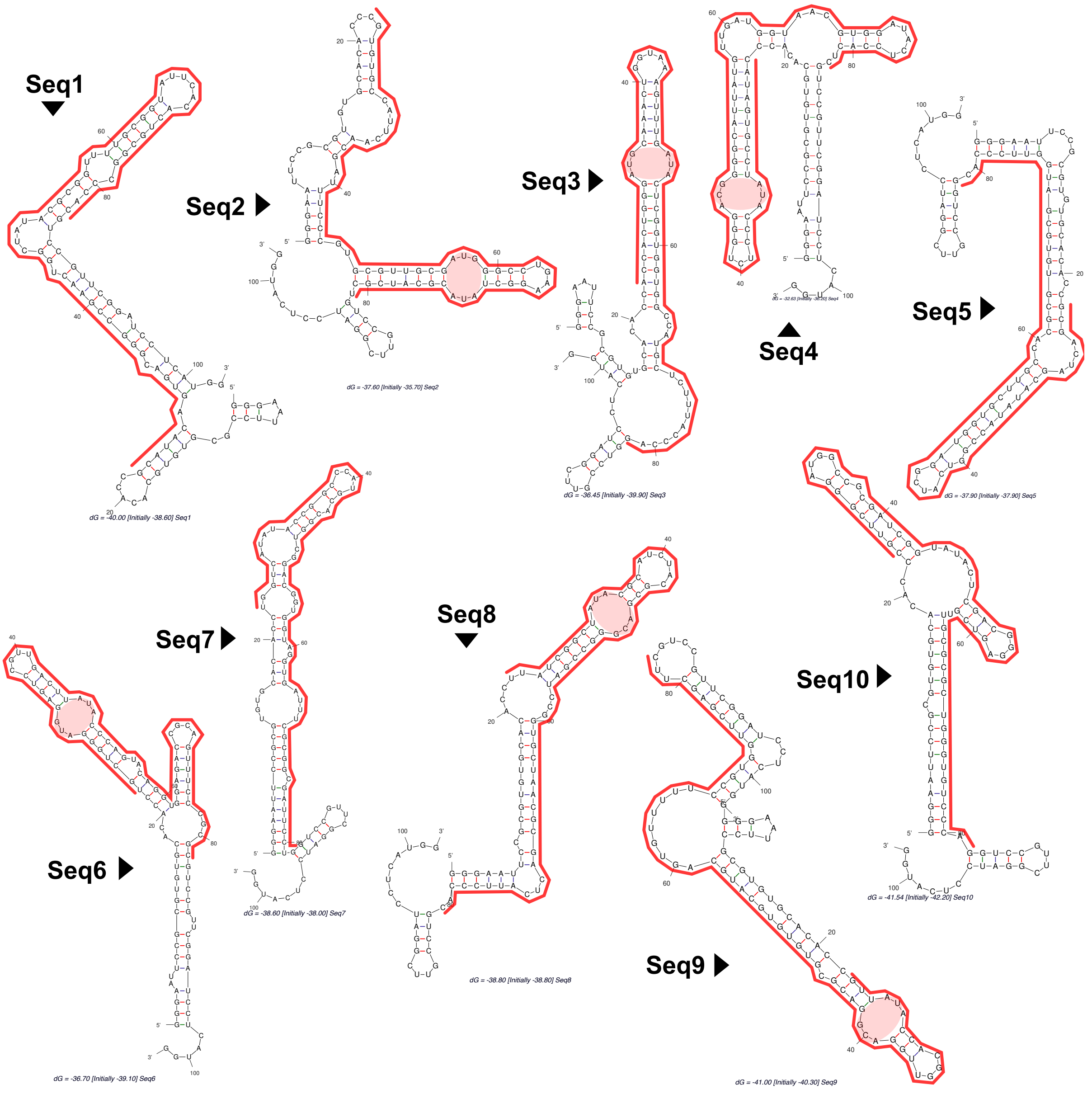

Clone	Optimal structure (Fig. 2)		Suboptimal structure-1 (Fig. S3)		Suboptimal structure-2	
	ΔG (kcal/mol)	AUA/AYG internal loop	ΔG(kcal/mol)	AUA/AYG internal loop	ΔG(kcal/mol)	AUA/AYG internal loop
**Seq1**	−40.8	+	−38.6	−	−38.6	+
**Seq2**	−37.8	+	−35.7	+	−34.5	+
**Seq3**	−41.1	+	−39.9	+	−38.4	+
**Seq4**	−37.3	+	−36.2	+	−35.4	+
**Seq5**	−38.5	+	−37.9	−	−36.7	+
**Seq6**	−39.6	+	−39.1	+	−37.9	+
**Seq7**	−39.8	+	−38.0	−	−36.9	−
**Seq8**	−41.9	+	−38.8	+	−38.3	+
**Seq9**	−40.7	+	−40.3	+	−38.5	+
**Seq10**	−45.7	+	−42.2	−	−41.2	−

Figure S2. Suboptimal foldings of Seq1–Seq10 predicted by mFold program, and their corresponding ΔG values.

A

Clones from the 12th library

The strand containing "ANA" (5'→3')	Count*1	The opposite strand (3'→5')	Count*1
UAUAC	23	GGUAG	13
GAUAC	9	GGCAG	12
UAAAC	2	CGUAG	6
UAUAA	1	AGUAG	1
UAUAU	3	GGCAU	1
UAUAG	2	GGCAA	3
CAUAC	1	CGCAG	3
		GGCAC	2
total	41	total	41

*1 : The count of clones among the 48 isolated clones.

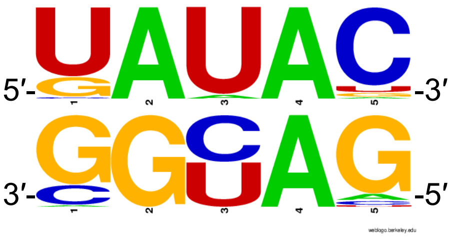

B

Clones from the 13th library

The strand containing "ANA" (5'→3')	Count*1	The opposite strand (3'→5')	Count*1
UAUAC	30	GGCAG	18
UACAC	1	GGUAG	14
GAUAC	3	GGGAG	1
GAGAC	1	CGUAG	2
UAAAC	1	CGCAG	2
AAUAC	1	AGUAG	1
UAGAC	2	UGCAG	1
total	39	total	39

*1 : The count of clones among the 48 isolated clones.

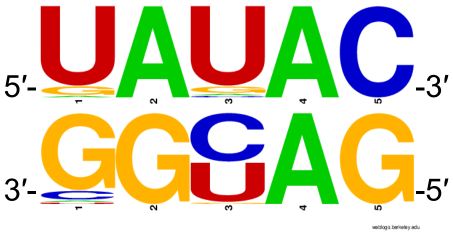

Figure S3. Number of clones containing the 5′-AUA-3′ sequence or the 5′-ABG-3′ (B = U, G, or C) sequence within the 3 × 3 internal loop. The clones were obtained from (A) the 12th library and (B) the 13th DNA library.

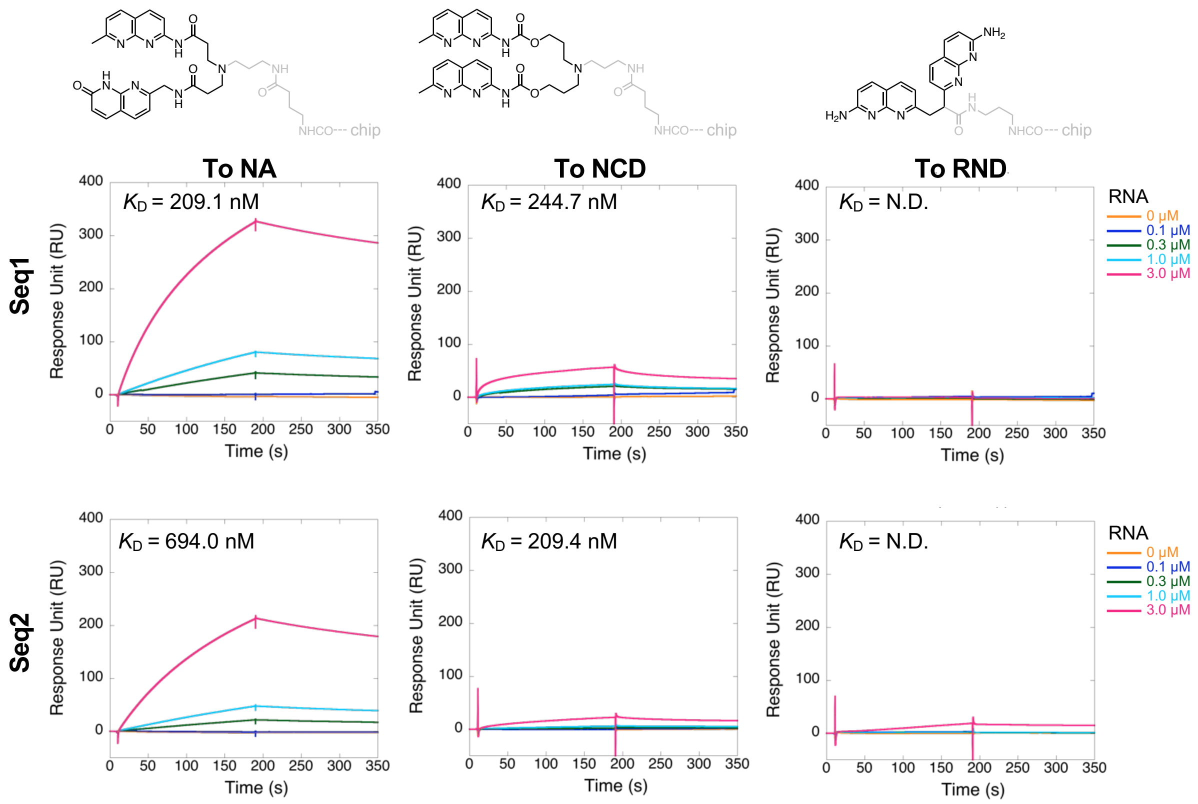

Figure S4. SPR analysis of binding of Seq1 and Seq2 to different small molecules. NA (naphthyridine-azaquinolone), NCD (naphthyridine carbamate dimer), and RND (restricted naphthyridine dimer) were immobilized on a SPR sensor chip by amine coupling. The immobilized amount of the small molecules were 101.8 RU for NA, 65.3 RU for NCD, and 622.2 RU for RND. Solution containing **Seq1** or **Seq2** with different concentrations were then flowed over the small-molecule immobilized sensor surface, and SPR signals were recorded.

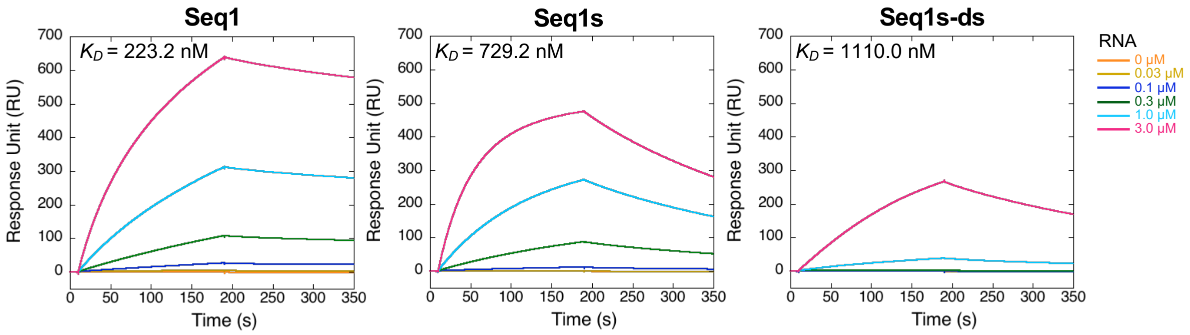

Figure S5. SPR analysis of binding of Seq1, Seq1s, and Seq1s-ds to NA. Solution containing **Seq1**, **Seq1s**, or **Seq1s-ds** with different concentrations were flowed over the NA immobilized sensor surface, and SPR signals were recorded.

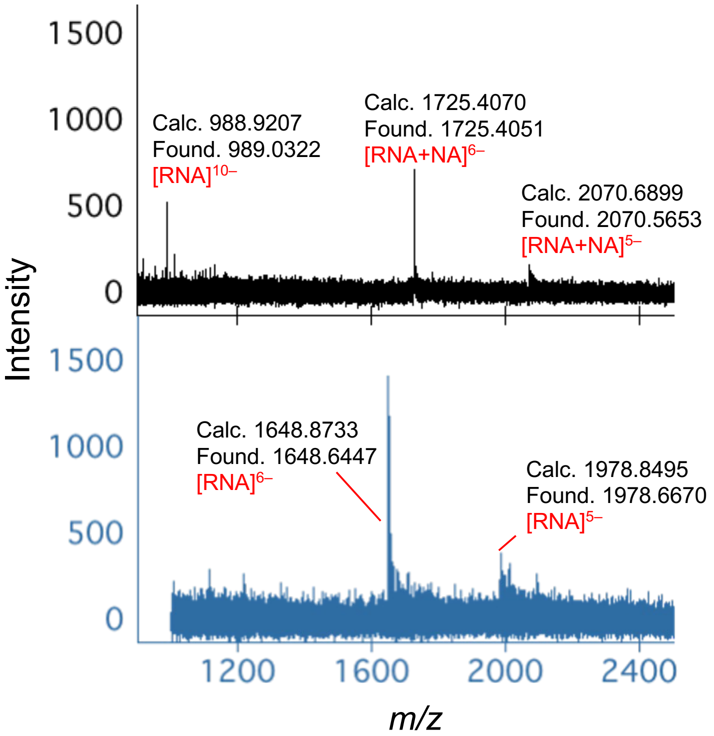

Figure S6. CSI-TOF mass spectra of Seq1s-NA complex. The CSI-TOF mass spectra of **Seq1s** in the absence (light blue) and presence (black) of NA. The samples were prepared as described in Material and Methods section.

A

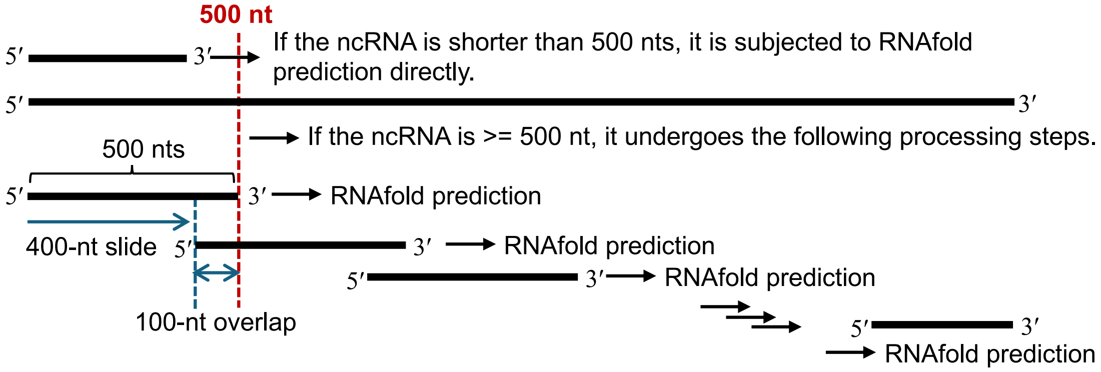

B

Motif	Count	Transcript ID
5′-GACGG-3′/5′-UAUAC-3′	3	>> ENST00000784640.1, ENST00000784641.1, ENST00000784646.1
5′-GAUGG-3′/5′-UAUAC-3′	4	>> ENST00000655645.1, ENST00000661392.1, ENST00000653715.1, ENST00000658271.1
5′-UAUAC-3′/5′-GACGG-3′	0	
5′-UAUAC-3′/5′-GAUGG-3′	2	>> ENST00000807793.1, ENST00000765360.1

C

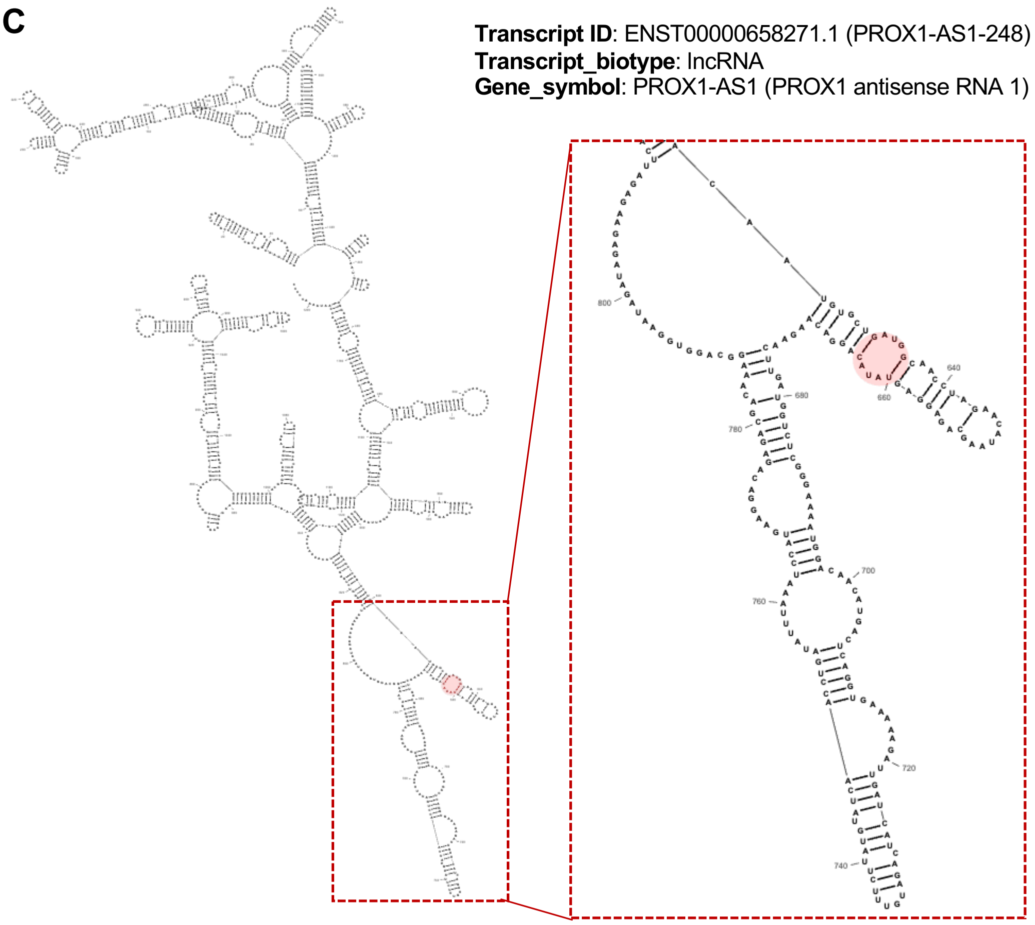

Figure S7. Motif search in ncRNA. (A) Workflow for secondary-structure prediction of non-coding RNAs using RNAfold. (B) Summary table of the identified internal loop motifs detected in ncRNA transcripts. The number of occurrences and corresponding Ensembl's transcript IDs are shown. (C) Predicted secondary structure of the transcript ENST00000658271.1 generated using RNAfold. The 3 × 3 internal loop is highlighted in red. Drawing of the predicted secondary structure was done using the web app "RNA canvas (https://rnacanvas.app/)".

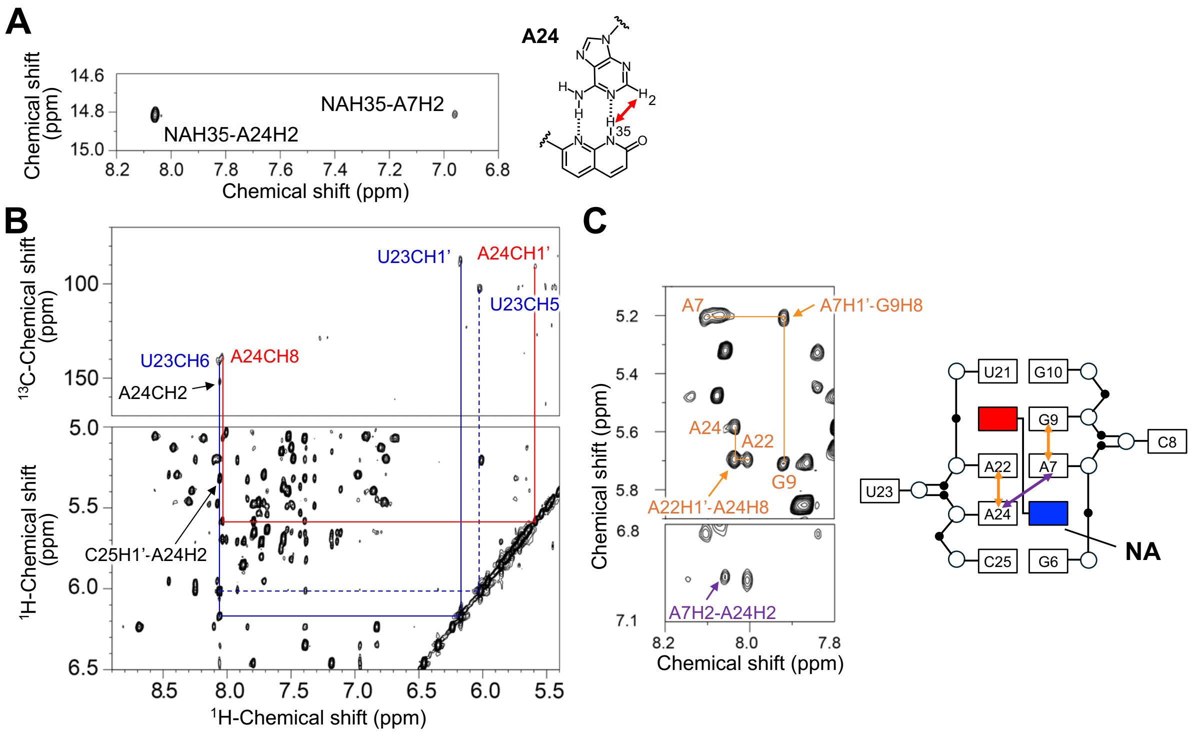

Figure S8. Supplementary NMR spectra for characterization of RNA and RNA-NA complex. (A) The intermolecular NOE signal between NA and A24. (B) NMR spectra of the residue-specific stable isotope labeled RNA. (C) NOE signals between A7/A22 and their adjacent residues (orange arrows) and between A7 and A24 (purple arrows). The red rectangle indicates the naphthyridine moiety, and the blue rectangle indicates the azaquinolone moiety in NA molecule.

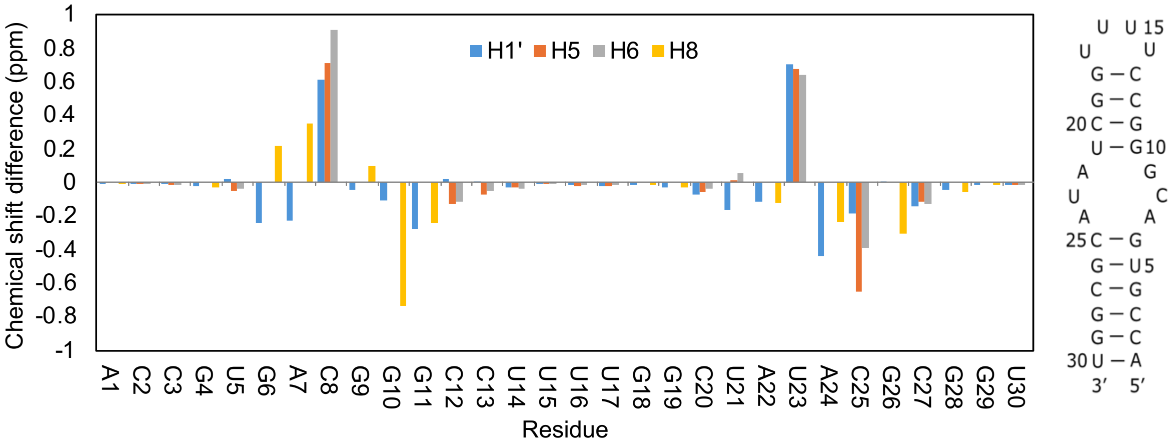

Figure S9. Chemical shift differences between free RNA and RNA-NA complex.

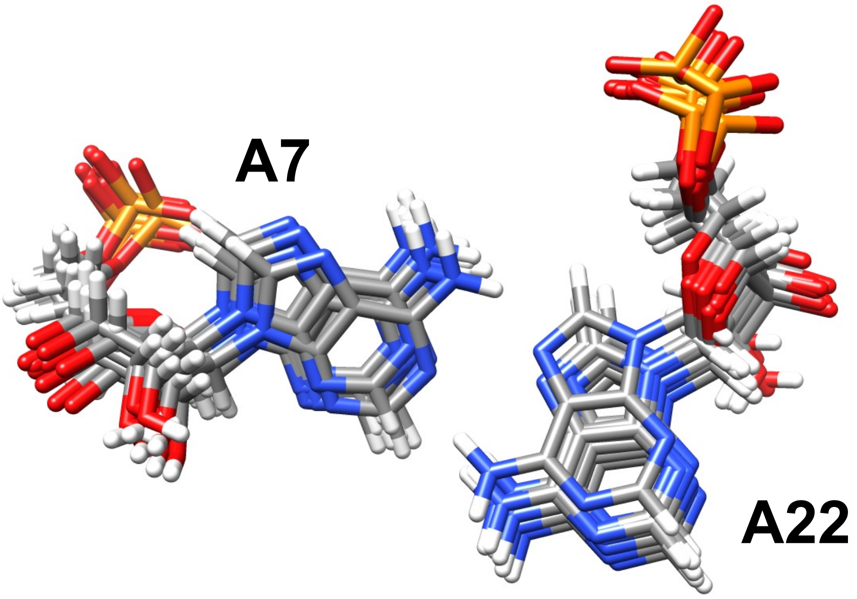

Figure S10. Close-up view of the base pairing between A7 and A22 residues

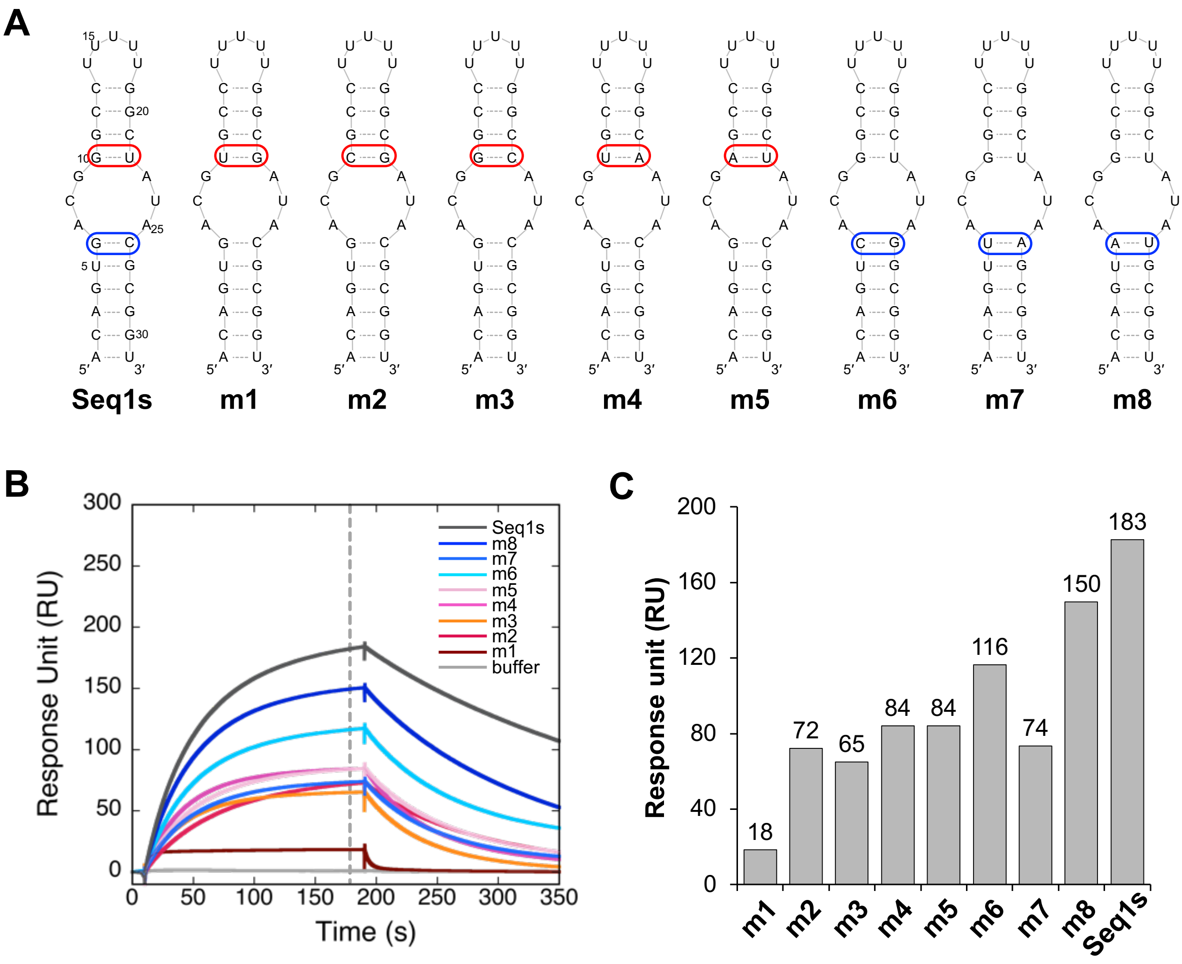

Figure S11. Effect of the difference in the flanking base pairs of the 3 × 3 internal loop on the interaction with NA. (A) Predicted secondary structures of the m1–m8 mutants. (B) SPR analysis of binding of m1–m8 mutants to NA. The RNA solution at 3 μM were flowed over the NA immobilized sensor surface, and SPR signals were recorded. (C) Bar graph showing values of response unit at steady state obtained for the binding of m1–m8 to NA.

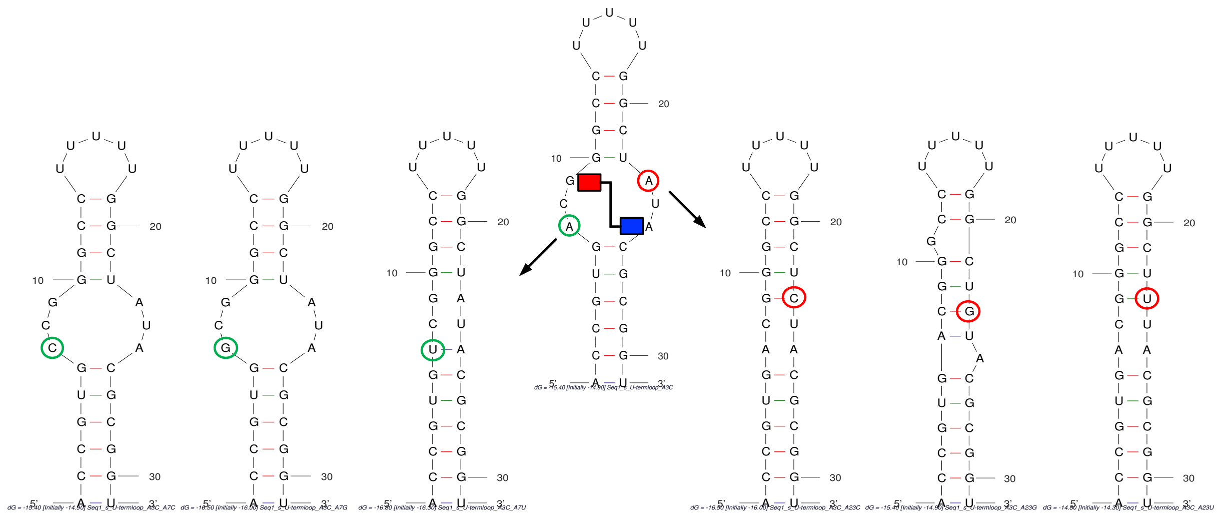

Figure S12. Predicted secondary structures of mutants of altered 3 × 3 internal loops. The secondary structure of the mutant, where the adenine not involved in binding to NA in the 3 × 3 internal loop was replaced with another base, was predicted using mFold. The blue and red rectangles connected by lines represent NA.

Table S1. The clones identified from the 13th library by next-generation sequencing

Reads from the 13th library

Ranking	Randomized region	Frequency[*1]
1	---- GCAUACAGUGACGGGCCGAACUGGCUAUACGCGGUUUUGCGGUAUUCACACUGCGGCCCAC ----	2.03% ⇒ Seq1
2	---- GCGACUAGCAUAUACCGGUCAUCGGAUGGUGCUUGCCACGCGUGUGCGAUGGUUCCCACG ----	1.54% ⇒ Seq5
3	---- UUAUCGGCUAUACGCAUCUACGCGACGGGCCGAUCCGGUGCUAACGCGACUCAUUCCCAC ----	1.25% ⇒ Seq8
4	---- GUUAUACCACGGUUGGACGGACGCGUGUGUGCAUGCAGUGUUUUUCCGUGGUUCGAGCUUC ----	0.92% ⇒ Seq9[*2]
5	---- CGUGUGCCAUUCAACGAUUUCCCGUGCGUUGCGAUGGGCCUGAAGGCUAUACGCAUCGCU ----	0.92% ⇒ Seq2
6	---- CGUUCGGGAUGGCCGCGAUCGGUAUACUCGACGGGAGUCGUUGCGCGCUGGGUGUCCCAG ----	0.83% ⇒ Seq10[*2]
7	---- UGGUCAUAUACCGGCCCAUGCACGGUCGGACGGUGGUAGGUGAUUUCGCGGCGAUUCCUG ----	0.78% ⇒ Seq7

[*1] : The frequency of each sequence in 66364 clustered reads.
[*2] : The sequences identified through next-generation sequencing analysis.

The enrichment of the clones identified by cloning and Sanger sequencing was further confirmed by next-generation sequencing among the top seven enriched sequences.

Table S2. The sequences of clones identified from 12th DNA library highlighting the conserved motif

RNA clones from 12th DNA library

Clone	Randomized region
**Seq2** ×3	CGUGUGCCAUUCAACGAUUUCCCGUGCGUUGCG**AUG**GCCUGAAGGCU**AUA**CGCAUCGCU
**Seq1** ×2	GCAUACAGUG**ACG**GCCGAACUGGCU**AUA**CGCGGUUUUGCGGUAUUCACACUGCGGCCCAC
**Seq3** ×2	ACCACUGGG**AUG**CAAACUGGUAAAGUUUG**AUA**CUCGGUGGUGGCCAUGCUCUUUACCCAG
**Seq6** ×1	UGCUGGG**AUG**GAGUCCGUUGACUU**AUA**CCCAGUACAGGUGGAGACCGCAGUUUCCCGCGC
**Seq7** ×1	UGGUCAU**AUA**CCGGCCCAUGCACGGUCGG**ACG**GUGGUAGGUGAUUUCGCGGCGAUUCCUG
The clones below were identified only once across the 12th and 13th DNA library.	
QC-2-8-001	GCUUGCAU**AAA**CGAAUUCUAUAUCG**ACG**GUGCACGCGGUGCUUCUCACUUGGAUUCCCAUG
QC-2-8-002*[1]	GUUGCAUCGUUUUACUUGUCCUUUCCAGGGAGGGCACCGUCACAUUUCGCGUAUUCUCAG
QC-2-8-005	ACCCCAAUG**AUG**GCCGUUGGU**AUA**CAUGGGGCAUGUAAGAUCAACGCGUGGAGUUCAAUG
QC-2-8-006	CUCGG**AUG**GGCCAUUAUGUCGCU**AUA**CCGUGGUGCAUGUGCACUCGUGGAAGUUCACGCU
QC-2-8-010	UGGUCGAGCU**AUA**CGCCAUACCAACGGCG**AUG**GGCUCGCUCCCUUUCGCGCGUUUUCCCU
QC-2-8-012	UUCGGCUG**AUG**GGUCGUCGUAAACGACU**AUA**CAGCGUUCUGUGUGCGGUCGUGCUUCCUCG
QC-2-8-013*[1]	GAUAGCUGGUCGUUUGGGAGUUGUGCCUUGCUUGUGCUCGCGUAGCUGACCCUUCGUUUC
QC-2-8-014	UGACGUG**AUG**ACAUCUGUCUCGAGACGAUGU**AUA**CACCUGAAUGCUCAGCGGUAGUCCCC
QC-2-8-015*[1]	CGUCUCUCACAGCAGUCGUGCAGUGGGGGAUGCUGAUGCCUUUGGUUGUGCCCAUCCCCUC
QC-2-8-016	CUACGUCCGAUGGG**AUA**CAUGGCAAGUCCAUG**AUG**CUCAUGGGAUUUUCAAAGCACACGCC
QC-2-8-017	UGGCAUGGCUU**AUA**AUGUUCGCCAU**ACG**GAGCUAUGCUAAUGUGUCACAGGCGUUCCUUC
QC-2-8-018	AUU**AUA**UCGUUGUGUUGCGA**ACG**GAUGGUGAACACGCACGCAGGAUUCCUUGUCCCUCCU
QC-2-8-019	GCAUAAGAGCUG**ACG**GGUUUCAGCU**AUA**CAGCUUUGCGUUGUGGUGUGUUUUUCUUCCUC
QC-2-8-020	AUUCCUGCAU**AUA**CGUCCGCGUCGACG**AUG**GUGUUGGAUGGUGGAAUUAGUGCGACUCCC
QC-2-8-021	ACAGGUGGCUG**ACG**GGUUUACU**AUA**CAGACCACCUGAGUGUUGGGCACGGAGGUCCCUUC
QC-2-8-023	AG**AUA**CGUAUCCGCG**ACG**CUGGCGUGUGCGUACGGAGUCAAUUGCAGCGUUUUGCUGUCG
QC-2-8-024	**ACG**GAGCUUAGCGUCUAAUGCUUGGGCUU**AUA**GGUGUAUACAUAUGUUGGAAUUUCUCAUC
QC-2-8-025	CGUU**AUA**CAACAGAUACUGUUG**AUG**GACGCGGUUGCGCAUUGCGCGUCUUCUCGACCUG
QC-2-8-026	GGUUUGGGG**ACG**GGCAAAUAUGCUU**AUA**CUCCAUGCCGGGAGUGCCGCUACUUUCCCUG
QC-2-8-027	CAUCUGGAU**AUA**CCCCACGCUCGGGG**AUG**GUCCAGACGGUAUGCAGGCAGGUACCCGUCG
QC-2-8-029	UUGUCAAGCUCGG**AUG**GCCGUUUUGGU**AUA**CCCUUGACUCGGUCUCACACGCCGCACACC
QC-2-8-030	AUGGGGGA**ACG**GCGGGCUUGGCUCGU**AUA**UCCCUCGCGGAAGUCUGCACCGUGUUUCCCU
QC-2-8-031	AUGGCG**AUG**CGUGAACACG**AUA**CGCCAGGUUGAUACUGCGCUUCUCUUGGACUUCUCUUCCU
QC-2-8-032*[2]	CGGACAGCCCGAUUCGAGCCAAAGUACGGCCACUU**AUA**CUCGCUGAAG**AUG**GAGUCGGCC
QC-2-8-033	CUGCUUCUGG**ACG**GUCGGUGAC**AUA**CCAGUGGCAGGGCGUACGCAGUUUUGGUACUCCCCC
QC-2-8-034	UCU**AUA**CGUGGCCGUUGCACCAUG**ACG**GGAGGCUGGAUUUCGGCACCGUGGACUCCCCAG
QC-2-8-036	CCCGUGCGUUACCGUCUUACUCUACUGGCGAAAGUCGACAUGUCGGCGUACUUCCGCCUG
QC-2-8-037	AAUCUGGGUCAGGCG**AUG**CCUUGGG**AUA**CGCUGCCCGGAUCCUGCCUUCGCGCGUCCCUUA
QC-2-8-038	GAGGUGUCCGG**ACG**CCCUUGG**AUA**CCGGCACCUUGGUUAUCAAGUGGAUCAUAUUCGU
QC-2-8-040*[1]	GGUAUAAGGCGCUACCUUCACCUUUUCUUGGGUGCAAUGUCUAUGUCAGUGUCAUUCCCC
QC-2-8-042	UACGGCCUU**AUA**GCGGUAAUCGC**ACG**GAGGCAGUUGUGCUGUGCUCACACACCAGUUCCC
QC-2-8-043	AAUGUCCACCGGUGCGG**AUG**CGCGUUGGCG**AUA**CCGUACCUAAGUCGUGGACACUUUUCCUC
QC-2-8-044*[1]	CCUGCCCUCGCUACAGCAGCCGUACCGUAGAACUGUACGACGUUUCCUCGUUUCCCUCCC
QC-2-8-045	GGUUUGGGG**ACG**GGCAAAUGCUU**AUA**CUCCAUGCCGGGAGUGCCGCUACUUUCCCUG
QC-2-8-047	AUAUGCUCG**ACG**GACCUUCAAUUAGUGGUU**AUA**CGAGUAUGUGCGUGUGCGCCGCGGGUC
QC-2-8-048	UAAGACCCCUCAGGCGUUUCGUGGAUGAU**AUA**CCUUCUGG**AUG**GUCAUCCUUUGGACCGCG
QC-2-9-097	GUGCGUGGCGCG**ACG**GAGUUAGCUU**AAA**CGCGCGCGUACGAUCACAUGCGCUCACUCCGC
QC-2-9-098	CAAGCCUGG**AUA**CACGAGUAGUG**ACG**CCAGGCAGGGUGAGGACAUACGUAACUCCCCCCU
QC-2-9-099	CACAUGGCA**ACG**GUCGGUUAGAU**AUA**UGCCUUCCGUGUGGUGUGACCGCGUCUUCCAAU

*[1]: The secondary structures of the sequences marked with an asterisk do not contain the conserved motif.
*[2]: Although this clone has "UAUAC" and "GAUGG" sequences, which were found in the conserved motif, the secondary structure predicted does not contain the motif.

In the table above, nucleotides where N is not U or B is not C/U in the 5′-ANA-3′/5′-ABG-3′ motif are highlighted in red.

Table S3. The sequences of clones identified from 13th DNA library highlighting the conserved motif

RNA clones from 13th DNA library

Clone	Randomized region
**Seq1 ×4**	GCAUACAGUG**AUG**GGCCGAACUGGCU**AUA**CGCGGUUUUGCGGUAUUCACACUGCGGCCCAC
**Seq4 ×2**	CAUAGUGCCU**AUA**CCCUCUGGG**AUG**GGCAUUAUGUUGAUGGUAACGUGGAUACUCCACUC
**Seq5 ×2**	GCGACUAGCAU**AUA**CCGGUCAUCGG**AUG**GUGCUUGCCACGCGUGUGCGAUGGUUCCCACG
**Seq8 ×2**	UUAUCGGCU**AUA**CGCAUCUACGCG**AUG**GGCCGAUCCGGUGCUAACGCGACUCAUUCCCAC
**Seq6 ×1**	UGCUGGG**AUG**GAGUCCGUUGACUU**AUA**CCCAGUACAGGUGGAGACCGCAGUUUCCCGCGC
**Seq7 ×1**	UGGUCAU**AUA**CCGGCCCAUGCACGGUCGG**AUG**GUGGUAGGUGAUUUCGCGGCGAUUCCUG
The clones below were identified only once across the 12th and 13th DNA library.	
QC-2-8-052	UGUGCUGGUAUCGU**AUA**CUCUUAACGGG**AUG**GCGAUACUUUAAUAAUAGCGGUUUCCCGC
QC-2-8-054	AUACG**AUG**GCUUUAGU**AUA**CGUAUGGUGCGCGUGUGCUGGCUUCCCCAACUUGUGUUCCC
QC-2-8-056	UGGCCU**ACA**CCCAGAGGG**AUG**GGGCCGGGCGAGUGCAUUUAUUCGCGGUCGUCUCGUUCG
QC-2-8-059	CAAACGCGUU**AUA**CAGCUGGUAGCUG**AGG**GAUCGUUGGGGCGCGCACACGACUUGUUCCC
QC-2-8-061*1	GCGACGCUGAAAAUGAUACGCGGGUGCUGAUACGACUAUUUCCCACGGCGUUUCUUUCGC
QC-2-8-064	GGCUUCCGUG**AUG**GGUGUCGACCACU**AUA**CACUGAAGCCGCUCACGCGCAAUGCCCCUU
QC-2-8-065*1	AGUGCAAGUUAACGUACUUCGAUGAACUUGAAAUAGGUGCGCACGUCCGCUACAGUUCUC
QC-2-8-066	UCAACCG**AUG**CGUUAUCG**AUA**CGGUGGGGUUUGAAUUACGUAUCUUCCCUUGAAUUCUCA
QC-2-8-068	CAGUAG**AUA**CACGCCAGUG**ACG**CUAUGGGUAGCGCACGAUACCCACCUUGCUCACGCGCA
QC-2-8-069*1	GCACUAUUGAUGGUGUGGCCUGUGCUCAUCCGGACCCGUAUC
QC-2-8-070*1	AUGAUCGUAGAUGGGAAACAUACUACGAUCGUAGGCUGCACUUCACGGAAUCUCGCAAUC
QC-2-8-071	GUUGUUCGG**AUG**CAUUUCGAUG**AGA**CCGCCGGUGGCAUACGGCCCGGUUCAUUCCUUCUUC
QC-2-8-076	CCCAGAAGAUGGG**AUG**GCGAAAGU**AAA**CCCAGUUCUGUCGGUGUAUUCCGCGCGAUUCUC
QC-2-8-077	U**AUA**CCCUUGAUGGG**AUG**GGGUGUGUAUUCGCUCGAAAGCUCGUAUC
QC-2-8-078	UGGCAUGCAU**AUA**CGGUGCAUCUGCCG**AUG**GUGCGUGUCGGACGUGCUGACUUUUUCCCGU
QC-2-8-079	G**AUG**GCGCAAGU**AUA**CGGAUGUGCUCGACGCGGACAUUUUCAUUAACGCGUUCUCCCACU
QC-2-8-081	ACAUUUGAUU**AUA**CAACAAUGACGUUG**AUG**GAUCUCAAGUGUGCAUGCGAUGGAGCUACC
QC-2-8-082	CGAUGUCAU**AUA**CAUCGAUUCGAUG**AUG**GUGCUUUCGUGAGUGCGCACAACGAUUUCCGC
QC-2-8-083*1	UGUCGGUGUAUCGCGCGACCUGCUUUUAGCAUGCGACAGCUUCUGGUUGUUCGAGCCCCUUC
QC-2-8-086	GAGACGCGAUG**AUG**AGCAAGCGCU**AUA**CAUCGGGUUUCGGUCCUUACGCUUGUUUCCUAA
QC-2-8-088	CACCGUGCAGU**AUA**CACGUCUUCCGUG**AUG**GCUGCCGCGGUCGUGCAAGCGAUCCCCACG
QC-2-8-089	UGGUCCG**AUG**GGGCGGUAGCCU**AUA**CGGAUCCAGGAUCUAUGGCGCGCACGGAUCCCGAU
QC-2-8-091	GGGUCGG**AUG**GCAUCAAGU**AUA**CUGAGUCGGCGUGUACGCUGUGUAAGGUUUCCCGCAUC
QC-2-8-092	UGGGAGGUCUGG**AUA**CCAGAUAUGG**ACG**CCAGUUCUCCCGCGCUGCACACUCCCAUCCCC
QC-2-8-093*1	CGCGGUCUGAACACCCUUCUCUAGGGAUGCGCGCGUUCGUGGGCACACCUCAUUCCUCUC
QC-2-8-094	UAUGUUCUGUG**ACG**UCGUGAGA**AUA**CGCAGUUCCGGUUGUGCGCUAUGGCAUGUCCUGCG
QC-2-8-095*1	CACCGUUCCAUCCUGCUCCUUGUAUCCGUGCAACUUAGGAGUUAGAAACGCGUUUCCCUG
QC-2-8-096	GACGUCG**AUG**GACUUGGUUAUUACCUUGUU**AUA**CUUCGCCGGUGGCACUCACUACUCCC
QC-2-9-107*1	GUCAUGGUCUCGUCGUGAUUUUUGUUAAGGUUCUCAGCUCUGAUUACUUCGCGGUUCCCC
QC-2-9-108	AUGCUCUGCG**AUG**GUCCCUUGUCGGAU**AUA**CGAGAGUGCCUCGGUGUGCUCAGUAUCCCAU
QC-2-9-110	GUCCUU**AGA**CAUGGCUACUCGUG**AUG**GAGGAUGGCUCUGUGCAUAACAACGUUUAUCCCUG
QC-2-9-111	CUCACGACUU**AUA**CCGUGUUUCGCGG**AUG**GAGCGGUGAGGUGUGCGUACACUCGGGUCCC
QC-2-9-112	UUAU**AUA**CCGCGGUUCGACAGGACCGCGG**AUG**GUUCGGUUGUGACGCUGUGUUCCUCGUG
QC-2-9-113	UGGCCCUUG**AUG**GACCAGAGGGGUU**AUA**CACGGGCCAGGUGCACCGUAGAACUCUCUCGC
QC-2-9-114	GCGAGGUUG**AUG**GGCCUUAGCU**AGA**CAAUCUCGUCUAGUGUACACGCCUGGUCCCGAAUC

*1: The secondary structures of the sequences marked with an asterisk do not contain the conserved motif.

In the table above, nucleotides where N is not U or B is not C/U in the 5′-ANA-3′/5′-ABG-3′ motif are highlighted in red.

Table S4. Chemical shifts of selected protons in free and NA-bound (1:1) RNA

	H1/H3			H1'			H5			H6			H8		
	Free	Bound	Δ(Bound-Free)	Free	Bound	Δ(Bound-Free)	Free	Bound	Δ(Bound-Free)	Free	Bound	Δ(Bound-Free)	Free	Bound	Δ(Bound-Free)
**A1**	-	-	-	5.958	5.953	-0.005	-	-	-	-	-	-	8.464	8.456	-0.008
**C2**	-	-	-	5.481	5.476	-0.005	5.302	5.298	-0.004	7.718	7.713	-0.005	-	-	-
**C3**	-	-	-	5.538	5.534	-0.004	5.478	5.464	-0.014	7.764	7.753	-0.011	-	-	-
**G4**	12.95	12.89	-0.06	5.689	5.667	-0.022	-	-	-	-	-	-	7.457	7.427	-0.03
**U5**	11.62	11.47	-0.15	5.307	5.329	0.022	5.424	5.373	-0.051	7.595	7.558	-0.037	-	-	-
**G6**	-	12.57	-	5.694	5.451	-0.243	-	-	-	-	-	-	7.621	7.842	0.221
**A7**	-	-	-	5.431	5.207	-0.224	-	-	-	-	-	-	7.748	8.104	0.356
**C8**	-	-	-	5.351	5.965	0.614	5.296	6.01	0.714	7.338	8.248	0.91	-	-	-
**G9**	-	11.01	-	5.755	5.711	-0.044	-	-	-	-	-	-	7.823	7.919	0.096
**G10**	-	9.54	-	5.764	5.656	-0.108	-	-	-	-	-	-	7.871	7.14	-0.731
**G11**	13.36	13.23	-0.13	5.657	5.384	-0.273	-	-	-	-	-	-	7.351	7.113	-0.238
**C12**	-	-	-	5.464	5.486	0.022	5.19	5.062	-0.128	7.632	7.52	-0.112	-	-	-
**C13**	-	-	-	5.49	5.495	0.005	5.467	5.398	-0.069	7.582	7.53	-0.052	-	-	-
**U14**	-	-	-	5.573	5.544	-0.029	5.662	5.635	-0.027	7.72	7.686	-0.034	-	-	-
**U15**	-	-	-	5.856	5.852	-0.004	5.866	5.857	-0.009	7.886	7.877	-0.009	-	-	-
**U16**	-	-	-	5.818	5.803	-0.015	5.731	5.71	-0.021	7.749	7.732	-0.017	-	-	-
**U17**	-	-	-	5.733	5.715	-0.018	5.71	5.69	-0.02	7.573	7.559	-0.014	-	-	-
**G18**	12.76	12.69	-0.07	5.719	5.708	-0.011	-	-	-	-	-	-	7.881	7.865	-0.016
**G19**	13.25	13.14	-0.11	5.845	5.82	-0.025	-	-	-	-	-	-	7.409	7.381	-0.028
**C20**	-	-	-	5.545	5.476	-0.069	5.185	5.13	-0.055	7.533	7.497	-0.036	-	-	-
**U21**	-	11.8	-	5.639	5.479	-0.16	5.642	5.653	0.011	7.737	7.79	0.053	-	-	-
**A22**	-	-	-	5.813	5.697	-0.116	-	-	-	-	-	-	8.128	8.006	-0.122
**U23**	-	-	-	5.465	6.168	0.703	5.339	6.018	0.679	7.419	8.059	0.64	-	-	-
**A24**	-	-	-	6.026	5.586	-0.44	-	-	-	-	8.025	-	8.268	8.037	-0.231
**C25**	-	-	-	5.502	5.32	-0.182	5.72	5.073	-0.647	7.783	7.393	-0.39	-	-	-
**G26**	10.99	10.99	0	5.486	5.491	0.005	-	-	-	-	-	-	7.621	7.314	-0.307
**C27**	-	-	-	5.462	5.32	-0.142	5.311	5.198	-0.113	7.611	7.483	-0.128	-	-	-
**G28**	12.48	12.4	-0.08	5.704	5.66	-0.044	-	-	-	-	-	-	7.447	7.388	-0.059
**G29**	13.42	13.41	-0.01	5.742	5.727	-0.015	-	-	-	-	-	-	7.197	7.181	-0.016
**U30**	-	-	-	5.771	5.757	-0.014	5.171	5.155	-0.016	7.588	7.575	-0.013	-	-	-

In the table above, data for the flipped-out nucleobases are highlighted in red.

Table S5. NMR restraints and statistics

Number of experimental restraints	
Distance restraints	440
Intra-residue (RNA)	136
Sequential (RNA)	100
Medium range (RNA)	15
Long range (RNA)	67
Inter-molecule	56
Intra-NA	34
Hydrogen bonding	32
Dihedral restraints	245
Planarity for base pairs	12
Heavy-atoms r.m.s. deviation (Å)[a]	
All	1.776 ± 0.665
All (pairwise)	2.065 ± 0.425
Backbone	1.323 ± 0.507
Backbone (pairwise)	1.380 ± 0.280
R.m.s.d. around the ideal values	
bonds (Å)	0.0030 ± 0.00005
angle (°)	0.7052 ± 0.0212

[a]Averaged r.m.s.d. between an average structure and the 10 converged structures were calculated. The converged structures did not contain experimental distance violation of >0.5 Å or dihedral violation >5°.

^{1}H-NMR spectra of Compound 5 (NA-linker)

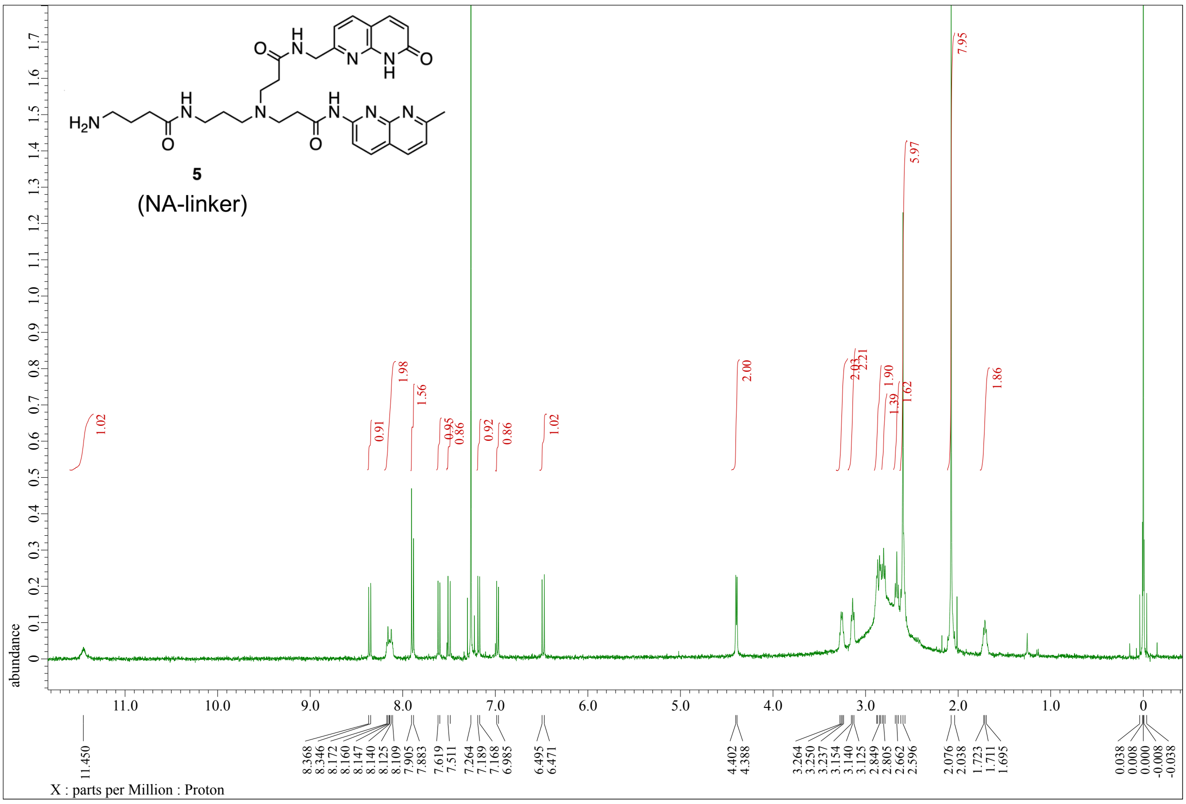

Mass spectra of Compound 5 (NA-linker)

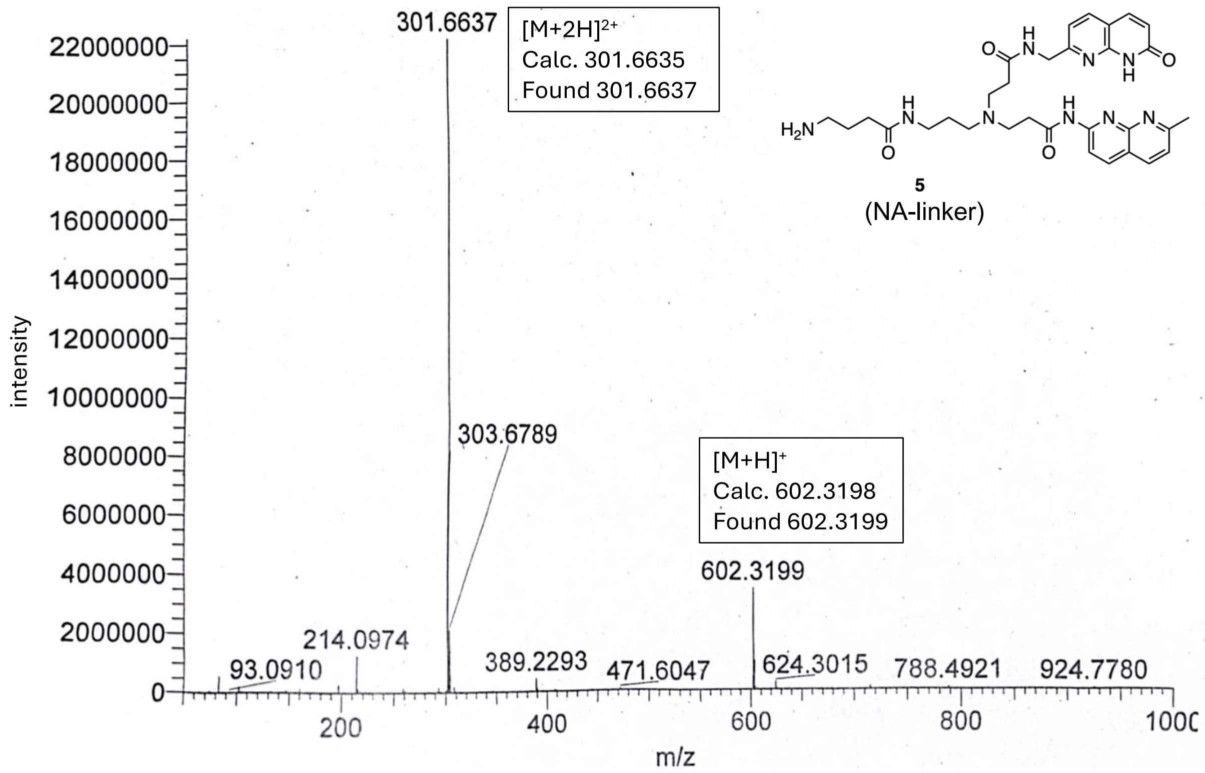

Supplement: SC-016-D5SC05255F-s001 [file SC-016-D5SC05255F-s001.pdf]
